# Cause-specific mortality in Korea during the first year of the COVID-19 pandemic

**DOI:** 10.4178/epih.e2022110

**Published:** 2022-11-23

**Authors:** Jinwook Bahk, Kyunghee Jung-Choi

**Affiliations:** 1 Department of Public Health, Keimyung University, Daegu, Korea; 2 Department of Environmental Medicine, Ewha Womans University College of Medicine, Seoul, Korea

**Keywords:** COVID-19, Mortality, Cause of death

## Abstract

**OBJECTIVES:**

This study aimed to examine the trends in total mortality between 1998 and 2020 and to compare the changes in a wide range of detailed causes of death between 2020 (i.e., during the coronavirus disease 2019 [COVID-19] pandemic) and the previous year in Korea.

**METHODS:**

We used registered population and mortality data for the years 1998–2020 obtained from Statistics Korea. The age-standardized all-cause mortality rate and the annual percent change between 1998 and 2020 were determined. The rate ratio and rate difference of the age-standardized mortality rate between 2019 and 2020 were calculated.

**RESULTS:**

The age-standardized all-cause mortality rate in Korea has been on a downward trend since 1998, and the decline continued in 2020. In 2020, 950 people died from COVID-19, accounting for 0.3% of all deaths. Mortality decreased for most causes of death; however, the number of deaths attributed to sepsis and aspiration pneumonia increased between 2019 and 2020 for both men and women. Age-specific mortality rates decreased or remained stable between 2019 and 2020 for all age groups, except women aged 25–29. This increase was mainly attributed to a higher number of suicide deaths.

**CONCLUSIONS:**

This study shed light on the issues of sepsis and aspiration pneumonia despite the successful response to COVID-19 in Korea in 2020. Cases of death from sepsis and aspiration pneumonia should be identified and monitored. In addition, it is necessary to develop a proactive policy to address suicide among young people, especially young women.

## GRAPHICAL ABSTRACT

[Fig f4-epih-44-e2022110]

## INTRODUCTION

It has been 3 years since coronavirus disease 2019 (COVID-19), which is caused by severe acute respiratory syndrome coronavirus 2 (SARS-CoV-2), first spread around the world. On January 30, 2020, the World Health Organization announced an international public health emergency following the initial detection of the disease in Wuhan, China, in December 2019. On February 29, 2020, it increased the global risk to “very high,” and on March 11, 2020, it declared the COVID-19 outbreak a pandemic [1]. As of July 27, 2022, a total of 570,005,017 people have been confirmed as infected, and 6,384,128 people have died [2]. New SARS-CoV-2 mutations have emerged. The COVID-19 pandemic is still going strong despite the development of COVID-19 vaccines and treatments. As of July 27, 2022, there have been 19,535,242 cases of COVID-19 in Korea and 24,957 deaths [3].

Korea’s response to COVID-19 has been successful without a nationwide lockdown [4]. Three infection crises occurred in February, August, and December 2020; however, they were all successfully resolved with a prompt government response, including diagnostic testing, contact tracing, timely treatment, quarantine, and social distancing [4,5]. Citizen cooperation was crucial to the success of the government’s quarantine efforts [6]. Vaccinations began in February 2021 [7]. This successful response has led to a low number of confirmed cases and deaths, and several studies have found that there were no excess deaths in Korea during the COVID-19 pandemic in 2020 [8–11].

Despite the excellent outcome of Korea’s quarantine measures, there have been reports of increased mortality due to specific causes or delayed treatment during the COVID-19 pandemic [12–15]. When mortality rates were analyzed by separating them by deaths in medical facilities and deaths outside of medical facilities, a significant increase in deaths outside of medical facilities was observed in 2020. This indicates potential changes in healthcare-seeking behaviors and the accessibility of emergency medical services during the epidemic [12]. A study conducted in Taiwan, where there were no excess deaths, reported fewer deaths from pneumonia and influenza in 2020 [16]. In contrast, mortality from pneumonia, cardiovascular disease, and diabetes increased between January and March 2020 in Wuhan, a location that reported excess deaths [17]. However, studies have had limitations in analyzing a small number of causes of death or using a broader classification of causes of death. This study aimed to examine the trends in total mortality between 1998 and 2020 and compare the changes in a wide range of detailed causes of death between 2020 during the COVID-19 pandemic and the previous year in Korea.

## MATERIALS AND METHODS

### Data

We used registered population and mortality data for the years 1998–2020. Both data sets are publicly accessible by visiting a website administered by Statistics Korea. The number of deaths was obtained from death certificate data provided by the MicroData Integrated Service ( https://mdis.kostat.go.kr/) of Statistics Korea [18]. Mortality data included gender, age, and cause of death. Causes of death were coded according to the International Classification of Diseases, 10th revision (ICD-10). The mid-year population data based on information from the resident registration system of the Korean government were obtained from the Korean Statistical Information Service (http://kosis.kr/) of Statistics Korea [19]. The registered population data provide the number of the population according to gender and one-year age groups. Supplementary Material 1 presents the population size and number of deaths during the study period.

We categorized the causes of death into 17 broad classes and 48 specific groups (Supplementary Material 2) based on the categorization of Statistics Korea. Furthermore, sub-specific causes (i.e., alcohol-specific disorders and poisonings, smoking-related causes, and avoidable causes including amenable and preventable causes) were categorized separately. We obtained the list of causes of death associated with smoking from a 2019 United States study [20]. The list of causes of death associated with alcohol-specific disorders and poisonings was obtained from Organization for Economic Cooperation and Development (OECD)/Eurostat lists [21]. OECD/Eurostat lists were also used to categorize the causes of death that were considered amenable and preventable (Supplementary Material 3).

### Statistical analysis

The rate of age-standardized all-cause mortality (per 100,000 people) and the annual percent change between 1998 and 2020 were determined. Age-standardized mortality rates and their 95% confidence intervals (CIs) according to the cause of death were calculated using direct standardization. The 2015 Korean population was used as the standard population. The rate ratio (RR) and rate difference (RD) of the age-standardized mortality rate were calculated as relative and absolute measures for differences between 2019 and 2020, respectively. Age-standardized mortality rates, RR, RD, and their 95% CIs were calculated using the PROC STDRATE procedure in SAS based on a normal distribution without bootstrap. We presented the age-standardized mortality rates by specific causes of death using 2015–2019 combined data and compared the age-standardized mortality rate between 2015–2019 and 2020 in Supplementary Materrials 3–5. All analyses were conducted separately for men and women using SAS version 9.4 (SAS Institute Inc., Cary, NC, USA)

### Ethics statement

This study was approved by the Institutional Review Board of the Ewha Womans University College of Medicine, Seoul, Korea (SEUMC 2022-08-044).

## RESULTS

**Figure 1:**
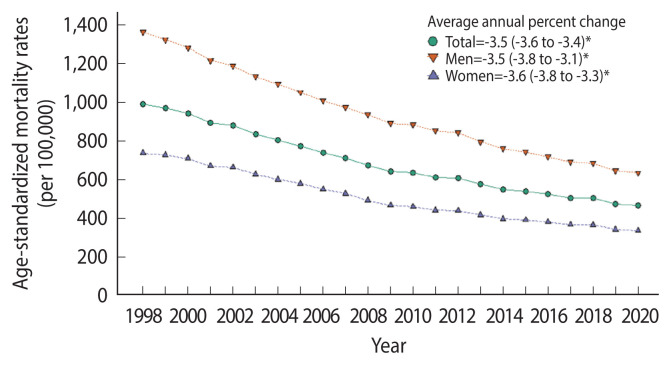
Trends of total mortality by gender among Korean in 1998–2020. *p<0.05.

In Korea, age-standardized all-cause mortality decreased from 1998–2020 (Figure 1). This trend was sustained in the first year of the COVID-19 pandemic. The age-standardized mortality rate per 100,000 people was 475.6 (95% CI, 473.8 to 477.3) in 2019 and 467.4 (95% CI, 465.7 to 469.1) in 2020. The decrease in mortality was greater among men than among women (Supplementary Material 1).

**Figure 2:**
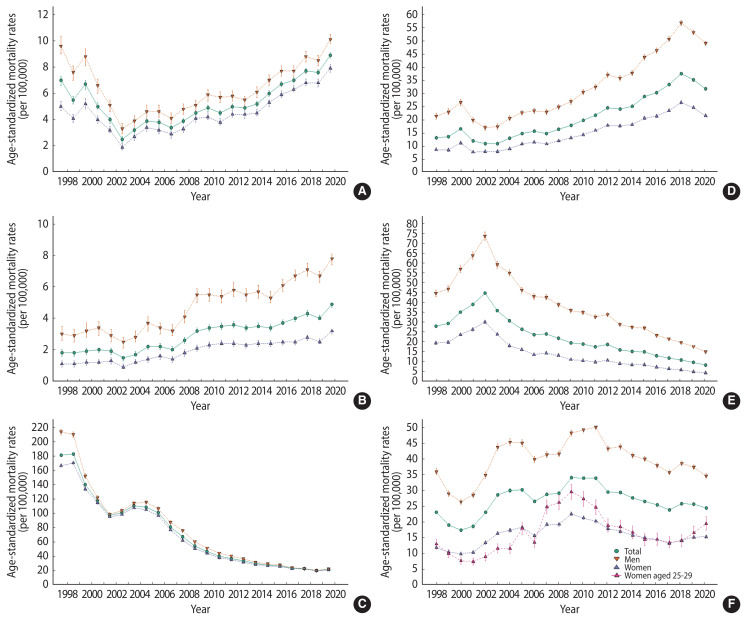
Trends of six cause-specific mortalities by gender among Korean in 1998–2020; (A) sepsis, (B) pneumonitis due to solids and liquids, (C) senility, (D) pneumonia, (E) chronic lower respiratory disease, and (F) suicide.

Tables 1–3 show the cause-specific mortality rates and their differences between 2019 and 2020. In 2020, there were 950 (483 men and 467 women) deaths due to COVID-19. COVID-19 deaths accounted for 0.3% of all-cause mortality in 2020. Mortality decreased for most causes of death; however, it increased for sepsis, pneumonitis due to solids and liquids, and senility between 2019 and 2020 (Figure 2A–C). Among them, except for senility, the mortality rates for sepsis and pneumonitis due to solids and liquids were higher than the average mortality rate in 2015–2019 (Supplementary Materials 3–5). This pattern was observed for both men and women. The number of deaths attributed to sepsis was 1,183 (540 men and 643 women), and the number of deaths attributed to pneumonitis due to solids and liquids was 751 (381 men and 370 women).

Among men, the mortality rate decreased the most for cancer, followed by respiratory disease and external death between 2019 and 2020. Respiratory disease, cardiovascular disease, and cancer showed the highest decrease in the mortality rate among women. Among the detailed causes, pneumonia decreased by 911 cases in 2020 compared to 2019, resulting in an RD of −3.4% (95% CI, −4.0 to −2.7), and chronic lower respiratory disease decreased by 508 people, resulting in an RD of −1.3% (95% CI, −1.6 to −1.0) (Supplementary Material 3). However, pneumonia showed a decreasing trend in 2019 and 2020 compared to 2018, and chronic lower respiratory disease also showed a decreasing pattern after 2002 (Figure 2D and E). Although the number of deaths attributed to smoking among those aged 35 and over increased, the age-standardized mortality rate decreased between 2019 and 2020. Mortality due to alcohol-specific disorders and poisonings increased for both men and women. Avoidable deaths, including those that were amenable and preventable, significantly decreased among men only.

**Figure 3:**
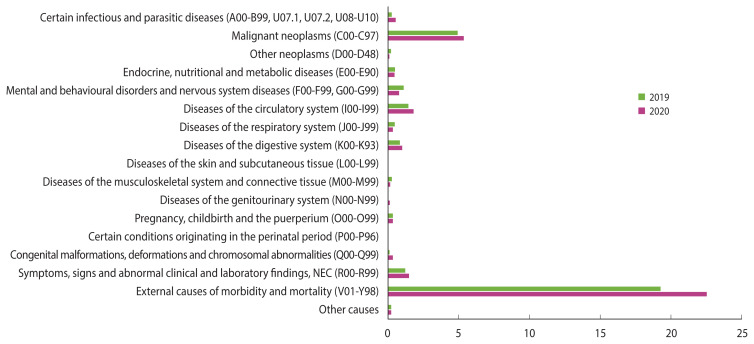
The age-specific mortality rate (per 100,000) among Korean women aged 25–29 according to causes of death in 2019 and 2020. NEC, not elsewhere classified.

Age-specific mortality rates were decreased or stable between 2019 and 2020 for all age groups, except for women aged 25–29 (Table 4 and Supplementary Material 6). The increase in mortality among women aged 25–29 was attributed to an increase in external causes of deaths; in particular, an increase in suicide deaths. The suicide rate among women aged 25–29 increased by 2.88 from 16.55 (per 100,000) in 2019 to 19.43 in 2020 (Figure 3 and Supplementary Material 7). Since 2017, there has been an increasing trend in the suicide rate for women aged 25–29 (Figure 2F).

## DISCUSSION

The age-standardized all-cause mortality in Korea has been on a downward trend since 1998 and this trend continued in 2020, the first year of the COVID-19 pandemic. In 2020, 950 people died from COVID-19, accounting for 0.3% of all deaths. Mortality was decreased for most causes of death; however, the number of deaths attributed to sepsis and pneumonitis due to solids and liquids increased between 2019 and 2020 for both men and women. Age-specific mortality rates decreased or were stable between 2019 and 2020 for all age groups, except for women aged 25–29. The increase in mortality among women aged 25–29 was mainly attributed to a higher number of suicide deaths.

On January 20, 2020, the first case of COVID-19 in Korea was officially confirmed by the Korea Centers for Disease Control and Prevention (hereafter “KCDC,”, the predecessor of the Korea Disease Control and Prevention Agency [KDCA], which was expanded, reorganized, and renamed on September 12, 2020) [5]. Korea had a successful COVID-19 response, including a testing-tracing-treatment (3T) strategy, swift application of focused, social distancing measures in high transmission areas, border control, and risk communication [4,6,22]. The cost of COVID-19 testing, quarantine, and treatment was covered by the Korean government and national health insurance program to reduce the economic burden [22]. Financial support for hospitalized or quarantined people was also provided [23]. These countermeasures led to a relatively low rate of confirmed cases and a low fatality rate, despite three COVID-19 waves in 2020 [24–26]. Based on the findings of this study, 950 deaths from COVID-19 were confirmed in 2020 death certificate data, accounting for only 0.3% of the total deaths. Along with the overall decrease in deaths attributed to other causes, the age-adjusted all-cause mortality rate in Korea decreased in 2020. This is in agreement with the findings of other studies analyzing the total mortality rate in Korea, which reported no excess deaths in 2020 [8,12].

However, a significant increase in death from sepsis may suggest the direct or indirect effects of COVID-19. The manifestations of COVID-19 range from asymptomatic to life-threatening sepsis [27], and sepsis findings were reported in all deceased patients in a previous study [28]. If death occurred after a COVID-19 polymerase chain reaction test was negative and the patient was transferred from the isolation ward to the general ward, the cause of death on the death certificate may only list sepsis without any mention of COVID-19 [29]. However, appropriate treatment for sepsis due to other causes may have been delayed or insufficient. The Korean government has prioritized medical resources for COVID-19 treatment to cope with the COVID-19 pandemic [30]. At the end of 2020, the government secured around 6,000 beds in infectious disease hospitals and supported the deployment of medical and paramedical personnel [31], which may have disrupted existing medical care. Delays in the management of patients with acute stroke or acute myocardial infarction during the pandemic were reported [14,15,32]. Additionally, longer or delayed emergency transfer times were noted [33]. In particular, in the case of fever with sepsis, emergency transportation was sometimes refused [34]. Therefore, it is necessary to monitor the occurrence of sepsis and associated death during the COVID-19 pandemic.

Cases of pneumonitis due to solids and liquids (i.e., aspiration pneumonia) also significantly increased. Approximately 5–15% of cases of community-acquired pneumonia may be attributed to aspiration pneumonia [35,36]; compared with other types of community-acquired pneumonia, aspiration pneumonia is associated with higher mortality [35]. The risk factors for aspiration pneumonia include impaired swallowing, degenerative neurologic diseases, impaired consciousness, poor dentition, and old age [35,37]. Aspiration pneumonia is a serious condition for those living in long-term care facilities, as these individuals are more likely to have risk factors [38,39]. Therefore, caregivers play a crucial role in preventing aspiration pneumonia [39–41]. As clusters of infections occurred in long-term care facilities among the elderly in the early stages of the COVID-19 pandemic, the government established guidelines for restricting visits (February 24, 2020) and eventually banned all visits (March 13, 2020) [42]. Cohort isolation was implemented when cases were confirmed in long-term care facilities for the elderly, and preventive cohort isolation was practiced in some places [43]. The quality of care for the elderly may have been reduced as the additional quarantine measures increased caregivers’ work burden and prevented family visits [42,43]. Moreover, similar to cases of sepsis, treatment for patients with fever associated with aspiration pneumonia may have been delayed [43]. A more thorough investigation of aspiration pneumonia deaths, taking into account the COVID-19 pandemic in 2021–2022, is required.

In this study, only the mortality rate for women aged 25–29 increased significantly in 2020 compared with 2019, which could be explained by a considerable increase in suicide deaths. However, the increase in the suicide rate of young Korean women is a trend that has continued since 2017 rather than a specific consequence of the 2020 COVID-19 pandemic, and it is not only limited to women aged 25–29 but also common among men and women in their 20s. The suicide rate from 2017 to 2020 in Korea increased from 16.7 (per 100,000) to 19.8 for men aged 20–24 and from 25.3 to 27.5 for men aged 25–29. The suicide rate of women was higher than that of men and increased from 9.5 (per 100,000) to 19.3 for women aged 20–24, and from 13.4 to 19.4 for women aged 25–29 [44]. Unemployment, job insecurity, financial hardships, social exclusion, and social isolation have been suggested as social risk factors for suicide among young adults [45,46]. Specifically, gender inequality and conflict may influence the suicide rate of young women [45]. However, the COVID-19 pandemic could also be related to suicide, as the risk of suicidal thoughts may have increased due to quarantine, exhaustion, social distancing, unemployment, and financial hardships [47]. Therefore, it is necessary to monitor changes in suicide patterns as long as the COVID-19 pandemic persists.

Senility, pneumonia, and chronic lower respiratory disease also showed significant changes in 2020 compared to 2019. However, when all of these diseases were analyzed by extending the period, it was difficult to judge that the change was a specific change from 2019 to 2020. However, since a decrease in pneumonia and chronic lower respiratory disease during the COVID-19 period has been reported in other studies [48], it is necessary to follow up in 2021 and 2022.

The study findings should be interpreted with caution due to certain limitations. First, death certificate data may be inaccurate, which could directly affect the accuracy of the study results. All deaths in Korea must be reported to the National Statistical Office within 1 month. When reporting the cause of death, death certification by a doctor is essential. In Korea, the rate of death registrations accompanied by a doctor’s medical certificate of death has continuously increased over the years, surpassing 90% since 2007, and after 2015—this study’s objective period—it exceeded 99% [49]. However, issues could arise due to the doctors’ lack of training and expertise in death certification [50]. A non-differential misclassification bias caused by the inaccuracies in recording the cause of death may not significantly affect the direction of annual comparison results, but it may have limitations in calculating an accurate mortality rate. Second, the projected mortality rate and the existing mortality rate may be compared to examine excess deaths; however, in this study, 2019 and 2020 were directly compared. For a more rigorous interpretation, we also analyzed whether the absolute death toll was relatively high and whether there were cases where the mortality rate exceeded the average between 2015 and 2019.

Despite its limitations, this study shed light on the issues of sepsis and aspiration pneumonia despite the successful response to COVID-19 in Korea. Cases of deaths from sepsis and aspiration pneumonia should be identified and monitored and preventive measures should be implemented. A proactive policy to address suicide among young people, especially young women, must be developed.

## Figures and Tables

**Figure f4-epih-44-e2022110:**
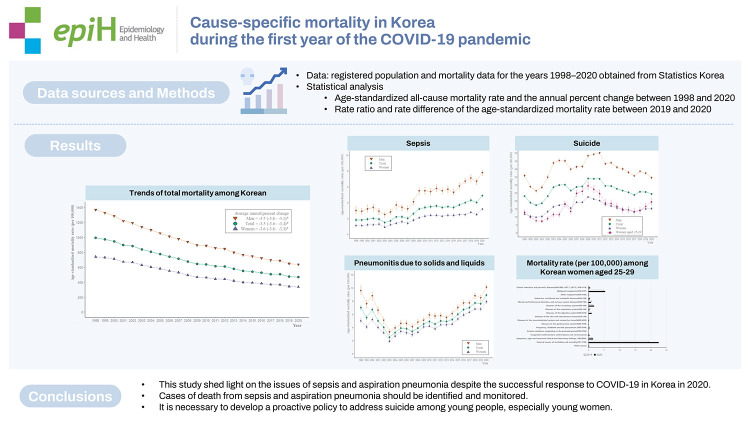


**Table 1:** Age-standardized mortality rates and differences between 2019 and 2020 by specific causes among Korean men and women

Causes	2019		2020		Between 2019 and 2020	
		
	No. of deaths	Age-standardized mortality rates (per 100,000)	No. of deaths	Age-standardized mortality rates (per 100,000)	No. of difference	RR (95% CI)
Certain infectious and parasitic diseases	8,692	13.7 (13.4–14.0)	10,419	15.5 (15.2–15.8)		1.13 (1.10, 1.16)
Tuberculosis	1,610	2.5 (2.4–2.7)	1,356	2.0 (1.9–2.1)	−254	0.81 (0.75, 0.87)
Sepsis	4,903	7.6 (7.4–7.8)	6,086	8.9 (8.7–9.1)	1,183	1.17 (1.13, 1.22)
COVID-19	-	-	950	1.4 (1.3–1.5)	950	-

Malignant neoplasms	81,203	134.0 (133.1–134.9)	82,204	130.1 (129.2–131.0)		0.97 (0.96, 0.98)
Esophageal cancer	1,554	2.6 (2.4–2.7)	1,564	2.5 (2.4–2.6)	10	0.96 (0.90, 1.03)
Stomach cancer	7,624	12.5 (12.3–12.8)	7,510	11.9 (11.6–12.1)	−114	0.95 (0.92, 0.98)
Colorectal cancer	8,966	14.6 (14.3–14.9)	8,944	13.9 (13.6–14.2)	−22	0.95 (0.92, 0.98)
Liver cancer	10,586	17.8 (17.4–18.1)	10,565	17.0 (16.7–17.3)	−21	0.96 (0.93, 0.98)
Gallbladder cancer	1,906	3.1 (2.9–3.2)	1,890	2.9 (2.8–3.0)	−16	0.95 (0.89, 1.01)
Biliary tract cancer	3,082	5.0 (4.8–5.1)	3,302	5.1 (4.9–5.2)	220	1.02 (0.97, 1.07)
Pancreatic cancer	6,396	10.5 (10.3–10.8)	6,775	10.7 (10.4–10.9)	379	1.01 (0.98, 1.05)
Lung cancer	18,574	30.3 (29.8–30.7)	18,673	29.1 (28.7–29.5)	99	0.96 (0.94, 0.98)
Breast cancer, female	2,622	4.6 (4.5–4.8)	2,725	4.7 (4.5–4.9)	103	1.02 (0.96, 1.07)
Ovarian cancer	1,234	2.1 (2.0–2.2)	1,369	2.3 (2.2–2.4)	135	1.08 (1.00, 1.16)
Prostate cancer	2,047	3.2 (3.1–3.4)	2,194	3.3 (3.1–3.4)	147	1.02 (0.96, 1.08)
Bladder cancer	1,550	2.4 (2.3–2.6)	1,593	2.4 (2.3–2.5)	43	0.98 (0.91, 1.05)
Non-Hodgkin’s lymphoma	2,015	3.3 (3.2–3.5)	2,069	3.3 (3.2–3.4)	54	0.99 (0.93, 1.05)
Leukemia	1,911	3.3 (3.1–3.4)	1,825	3.0 (2.9–3.2)	−86	0.93 (0.87, 0.99)

Endocrine, nutritional and metabolic diseases	9,502	15.2 (14.9–15.5)	10,052	15.3 (15.0–15.6)		1.01 (0.98, 1.03)
Diabetes mellitus	8,102	12.9 (12.6–13.2)	8,456	12.8 (12.5–13.1)	354	0.99 (0.96, 1.02)

Mental and behavioral disorders and nervous system diseases	17,575	27.2 (26.8–27.6)	18,138	26.5 (26.1–26.9)		0.97 (0.95, 0.99)
Dementia	1,0357	15.3 (15.0–15.6)	10,641	14.6 (14.3–14.9)	284	0.95 (0.93, 0.98)
Alcoholism	916	1.6 (1.5–1.8)	1,089	1.9 (1.8–2.0)	173	1.16 (1.06, 1.26)
Parkinson’s disease	3,476	5.4 (5.2–5.6)	3,571	5.3 (5.1–5.4)	95	0.98 (0.93, 1.02)

Diseases of the circulatory system	60,252	94.8 (94.0–95.6)	62,196	92.5 (91.8–93.3)		0.98 (0.96, 0.99)
Hypertensive diseases	5,631	8.5 (8.2–8.7)	6,100	8.6 (8.3–8.8)	469	1.01 (0.98, 1.05)
Ischemic heart diseases	13,699	21.8 (21.5–22.2)	14,056	21.3 (21.0–21.7)	357	0.98 (0.95, 1.00)
Heart failure	6,758	10.2 (10.0–10.4)	7,256	10.2 (10.0–10.4)	498	1.00 (0.97, 1.03)
Cerebrovascular diseases	21,586	34.2 (33.8–34.7)	21,860	32.8 (32.4–33.3)	274	0.96 (0.94, 0.98)
Hemorrhagic stroke	6,942	11.5 (11.3–11.8)	7,092	11.3 (11.0–11.6)	150	0.98 (0.95, 1.01)
Ischemic stroke	7,103	10.9 (10.7–11.2)	7,414	10.7 (10.5–11.0)	311	0.98 (0.95, 1.01)
Other stroke	7,541	11.8 (11.5–12.0)	7,354	10.8 (10.6–11.1)	−187	0.92 (0.89, 0.95)

Diseases of the respiratory system	36,655	56.2 (55.6–56.8)	36,368	52.5 (52.0–53.1)		0.94 (0.92, 0.95)
Pneumonia	23,168	35.3 (34.8–35.7)	22,257	31.9 (31.5–32.4)	−911	0.90 (0.89, 0.92)
Chronic lower respiratory diseases	6,176	9.5 (9.3–9.7)	5,668	8.2 (8.0–8.4)	−508	0.86 (0.83, 0.90)
Pneumonitis due to solids and liquids	2,631	4.0 (3.9–4.2)	3,382	4.9 (4.7–5.0)	751	1.21 (1.15, 1.27)
Interstitial pulmonary diseases	1,813	2.9 (2.8–3.1)	1,806	2.8 (2.6–2.9)	−7	0.95 (0.89, 1.01)

Diseases of the digestive system	11,963	20.0 (19.6–20.4)	12,870	20.7 (20.3–21.0)		1.03 (1.01, 1.06)
Liver diseases	6,496	11.4 (11.1–11.7)	6,979	11.9 (11.6–12.2)	483	1.05 (1.01, 1.08)
Alcoholic liver disease	3,658	6.6 (6.4–6.8)	3,941	7.0 (6.8–7.2)	283	1.05 (1.01, 1.10)
Liver cirrhosis	2,133	3.6 (3.4–3.8)	2,202	3.6 (3.5–3.8)	69	1.00 (0.94, 1.06)

Diseases of the genitourinary system	8,565	13.3 (13.1–13.6)	9,343	13.7 (13.5–14.0)		1.03 (1.00, 1.06)
Renal failure	6,036	9.5 (9.2–9.7)	6,589	9.8 (9.6–10.0)	553	1.03 (1.00, 1.07)

Symptoms, signs and abnormal clinical and laboratory findings	28,176	43.7 (43.2–44.2)	31,801	46.2 (45.7–46.8)		1.06 (1.04, 1.07)
Senility	13,522	19.8 (19.5–20.1)	15,823	21.4 (21.0–21.7)	2,301	1.08 (1.06, 1.11)
Other ill-defined and unspecified causes of mortality	8,549	14.3 (14.0–14.6)	9,143	14.5 (14.2–14.8)	594	1.02 (0.99, 1.05)

External causes of morbidity and mortality	27,282	48.5 (48.0–49.1)	26,442	46.1 (45.5–46.6)		0.95 (0.93, 0.97)
Transport accidents	4,221	7.4 (7.2–7.7)	3,947	6.8 (6.6–7.0)	−274	0.91 (0.87, 0.95)
Falls	2,665	4.5 (4.3–4.7)	2,663	4.3 (4.1–4.4)	−2	0.95 (0.90, 1.00)
Intentional self-harm	13,799	25.6 (25.2–26.0)	13,195	24.4 (24.0–24.8)	−604	0.95 (0.93, 0.98)

Sub-specific causes						
Alcohol-specific disorders and poisonings	4,725	8.5 (8.3–8.8)	5,191	9.2 (8.9–9.4)	466	1.08 (1.03, 1.12)
Smoking-related causes (+35 yr)	150,707	240.2 (239.0–241.4)	151,728	230.0 (228.8–231.1)	1,021	0.96 (0.95, 0.96)
Avoidable causes (0–74 yr)	81,553	147.0 (146.0–148.0)	81,373	142.4 (141.4–143.4)	−180	0.97 (0.96, 0.98)
Amenable causes	32,536	58.2 (57.6–58.8)	32,843	56.7 (56.1–57.3)	307	0.97 (0.96, 0.99)
Preventable causes	65,791	118.6 (117.7–119.5)	65,254	114.4 (113.5–115.3)	−537	0.96 (0.95, 0.98)

COVID-19, coronavirus disease 2019; RR, rate ratio; CI, confidence interval.

**Table 2:** Age-standardized mortality rates and differences between 2019 and 2020 by specific causes among Korean men

Causes	2019		2020		Between 2019 and 2020	
		
	No. of deaths	Age-standardized mortality rates (per 100,000)	No. of deaths	Age-standardized mortality rates (per 100,000)	No. of difference	RR (95% CI)
Certain infectious and parasitic diseases	4,103	17.0 (16.4–17.5)	4,890	19.2 (18.6–19.7)		1.13 (1.08, 1.18)
Tuberculosis	977	4.1 (3.8–4.3)	842	3.3 (3.1–3.5)	−135	0.82 (0.74, 0.90)
Sepsis	2,013	8.5 (8.1–8.9)	2,553	10.1 (9.8–10.5)	540	1.19 (1.12, 1.26)
COVID-19	-	-	483	1.9 (1.7–2.1)	483	-

Malignant neoplasms	50,281	196.7 (195.0–198.5)	50,817	189.6 (188.0–191.3)		0.96 (0.95, 0.98)
Esophageal cancer	1,425	5.4 (5.1–5.7)	1,404	5.1 (4.8–5.4)	−21	0.94 (0.88, 1.02)
Stomach cancer	4,956	19.5 (18.9–20.0)	4,807	17.9 (17.4–18.5)	−149	0.92 (0.89, 0.96)
Colorectal cancer	5,065	20.0 (19.4–20.6)	5,060	18.9 (18.4–19.4)	−5	0.94 (0.91, 0.98)
Liver cancer	7,784	29.4 (28.8–30.1)	7,812	28.5 (27.8–29.1)	28	0.97 (0.94, 1.00)
Gallbladder cancer	805	3.2 (2.9–3.4)	838	3.1 (2.9–3.3)	33	0.99 (0.90, 1.09)
Biliary tract cancer	1,763	7.0 (6.7–7.3)	1,946	7.3 (7.0–7.6)	183	1.05 (0.98, 1.12)
Pancreatic cancer	3,424	13.1 (12.7–13.5)	3,452	12.6 (12.2–13.0)	28	0.96 (0.92, 1.01)
Lung cancer	13,698	53.8 (52.9–54.7)	13,824	51.7 (50.9–52.6)	126	0.96 (0.94, 0.98)
Prostate cancer	2,047	8.7 (8.3–9.1)	2,194	8.8 (8.4–9.1)	147	1.01 (0.95, 1.07)
Bladder cancer	1,167	4.9 (4.6–5.2)	1,235	4.8 (4.6–5.1)	68	0.99 (0.92, 1.08)
Non-Hodgkin’s lymphoma	1,169	4.6 (4.3–4.8)	1,192	4.5 (4.2–4.7)	23	0.98 (0.90, 1.06)
Leukemia	1,143	4.4 (4.2–4.7)	1,048	3.9 (3.7–4.1)	−95	0.88 (0.81, 0.96)

Endocrine, nutritional and metabolic diseases	4,817	19.3 (18.7–19.8)	5,105	19.5 (19.0–20.0)		1.01 (0.97, 1.05)
Diabetes mellitus	4,121	16.5 (16.0–17.0)	4,320	16.5 (16.0–17.0)	199	1.00 (0.96, 1.04)

Mental and behavioral disorders and nervous system diseases	7,135	29.9 (29.2–30.7)	7,576	29.9 (29.2–30.6)		1.00 (0.97, 1.03)
Dementia	3,114	14.1 (13.6–14.6)	3,329	13.9 (13.5–14.4)	215	0.99 (0.94, 1.04)
Alcoholism	825	3.0 (2.8–3.2)	969	3.4 (3.2–3.7)	144	1.14 (1.04, 1.25)
Parkinson’s disease	1,549	6.5 (6.2–6.8)	1,643	6.5 (6.1–6.8)	94	0.99 (0.93, 1.07)

Diseases of the circulatory system	28,672	117.7 (116.3–119.0)	29,568	115.1 (113.7–116.4)		0.98 (0.96, 0.99)
Hypertensive diseases	1,795	7.8 (7.4–8.2)	1,997	8.1 (7.8–8.5)	202	1.04 (0.97, 1.11)
Ischemic heart diseases	7,696	31.0 (30.3–31.7)	7,959	30.4 (29.7–31.1)	263	0.98 (0.95, 1.01)
Heart failure	2,268	9.9 (9.5–10.3)	2,420	9.9 (9.5–10.3)	152	1.00 (0.95, 1.06)
Cerebrovascular diseases	10,626	43.5 (42.6–44.3)	10,630	41.2 (40.5–42.0)	4	0.95 (0.92, 0.98)
Hemorrhagic stroke	3,561	14.0 (13.6–14.5)	3,534	13.4 (12.9–13.8)	−27	0.95 (0.91, 1.00)
Ischemic stroke	3,435	14.6 (14.1–15.1)	3,614	14.4 (13.9–14.9)	179	0.99 (0.94, 1.03)
Other stroke	3,630	14.8 (14.4–15.3)	3,482	13.5 (13.0–14.0)	−148	0.91 (0.87, 0.95)

Diseases of the respiratory system	20,513	89.0 (87.8–90.3)	20,671	83.5 (82.3–84.6)		0.94 (0.92, 0.96)
Pneumonia	12,157	53.3 (52.4–54.3)	12,085	49.2 (48.3–50.0)	−72	0.92 (0.90, 0.95)
Chronic lower respiratory diseases	4,055	17.6 (17.0–18.1)	3,678	14.9 (14.4–15.3)	−377	0.85 (0.81, 0.88)
Pneumonitis due to solids and liquids	1,525	6.7 (6.3–7.0)	1,906	7.8 (7.4–8.1)	381	1.16 (1.09, 1.24)
Interstitial pulmonary diseases	1,180	4.7 (4.4–5.0)	1,185	4.5 (4.2–4.8)	5	0.96 (0.88, 1.04)

Diseases of the digestive system	7,316	28.2 (27.5–28.8)	7,922	29.4 (28.8–30.1)		1.04 (1.01, 1.08)
Liver diseases	4,772	17.8 (17.3–18.3)	5,192	18.9 (18.4–19.4)	420	1.06 (1.02, 1.11)
Alcoholic liver disease	3,140	11.6 (11.2–12.0)	3,354	12.0 (11.6–12.5)	214	1.04 (0.99, 1.09)
Liver cirrhosis	1,248	4.7 (4.4–5.0)	1,333	4.9 (4.6–5.2)	85	1.04 (0.97, 1.13)

Diseases of the genitourinary system	3,712	15.6 (15.1–16.1)	4,113	16.3 (15.8–16.8)		1.04 (1.00, 1.09)
Renal failure	2,990	12.5 (12.0–12.9)	3,323	13.0 (12.6–13.5)	333	1.05 (1.00, 1.10)

Symptoms, signs and abnormal clinical and laboratory findings	12,542	52.9 (51.9–53.8)	14,135	56.4 (55.5–57.4)		1.07 (1.04, 1.09)
Senility	4,210	19.8 (19.2–20.4)	5,150	22.2 (21.6–22.8)	940	1.12 (1.08, 1.17)
Other ill-defined and unspecified causes of mortality	5,391	21.0 (20.5–21.6)	5,602	21.1 (20.5–21.6)	211	1.00 (0.96, 1.04)

External causes of morbidity and mortality	18,896	73.1 (72.0–74.1)	18,032	68.2 (67.2–69.2)		0.93 (0.91, 0.95)
Transport accidents	3,162	12.1 (11.7–12.6)	2,955	11.0 (10.6–11.4)	−207	0.91 (0.86, 0.95)
Falls	1,928	7.5 (7.2–7.8)	1,926	7.2 (6.8–7.5)	−2	0.96 (0.90, 1.02)
Intentional self-harm	9,730	37.3 (36.6–38.1)	9,093	34.5 (33.8–35.3)	−637	0.93 (0.90, 0.95)

Sub-specific causes						
Alcohol-specific disorders and poisonings	4,084	15.0 (14.6–15.5)	4,459	16.0 (15.5–16.5)	375	1.06 (1.02, 1.11)
Smoking-related causes (+35 yr)	86,831	351.1 (348.8–353.5)	87,599	335.5 (333.2–337.7)	768	0.96 (0.95, 0.96)
Avoidable causes (0–74 yr)	58,665	214.2 (212.5–215.9)	58,339	206.2 (204.5–207.9)	−326	0.96 (0.95, 0.97)
Amenable causes	20,793	75.8 (74.8–76.9)	20,999	73.6 (72.6–74.6)	206	0.97 (0.95, 0.99)
Preventable causes	49,725	181.4 (179.8–183.0)	49,148	174.0 (172.4–175.5)	−577	0.96 (0.95, 0.97)

COVID-19, coronavirus disease 2019; RR, rate ratio; CI, confidence interval.

**Table 3:** Age-standardized mortality rates and differences between 2019 and 2020 by specific causes among Korean women

Causes	2019		2020		Between 2019 and 2020	
		
	No. of deaths	Age-standardized mortality rates (per 100,000)	No. of deaths	Age-standardized mortality rates (per 100,000)	No. of difference	RR (95% CI)
Certain infectious and parasitic diseases	4,589	11.1 (10.7–11.4)	5,529	12.6 (12.3–13.0)		1.14 (1.10, 1.19)
Tuberculosis	633	1.5 (1.4–1.6)	514	1.2 (1.1–1.3)	−119	0.78 (0.69, 0.88)
Sepsis	2,890	6.8 (6.5–7.1)	3,533	7.9 (7.6–8.2)	643	1.16 (1.10, 1.22)
COVID-19	-	-	467	1.1 (1.0–1.2)	467	-

Malignant neoplasms	30,922	89.3 (88.3–90.3)	31,387	87.5 (86.5–88.5)		0.98 (0.96, 1.00)
Esophageal cancer	129	0.4 (0.3–0.4)	160	0.5 (0.4–0.5)	31	1.22 (0.96, 1.54)
Stomach cancer	2,668	7.5 (7.2–7.8)	2,703	7.4 (7.1–7.7)	35	0.99 (0.94, 1.05)
Colorectal cancer	3,901	10.6 (10.3–11.0)	3,884	10.1 (9.8–10.4)	−17	0.95 (0.91, 1.00)
Liver cancer	2,802	8.0 (7.7–8.3)	2,753	7.5 (7.2–7.8)	−49	0.93 (0.89, 0.99)
Gallbladder cancer	1,101	3.0 (2.8–3.2)	1,052	2.8 (2.6–2.9)	−49	0.92 (0.85, 1.01)
Biliary tract cancer	1,319	3.5 (3.3–3.7)	1,356	3.4 (3.2–3.6)	37	0.98 (0.91, 1.06)
Pancreatic cancer	2,972	8.4 (8.1–8.7)	3,323	9.0 (8.7–9.3)	351	1.07 (1.02, 1.13)
Lung cancer	4,876	13.5 (13.1–13.9)	4,849	12.9 (12.5–13.2)	−27	0.95 (0.92, 0.99)
Breast cancer	2,622	8.9 (8.6–9.3)	2,725	9.1 (8.7–9.4)	103	1.02 (0.96, 1.07)
Ovarian cancer	1,234	4.0 (3.8–4.2)	1,369	4.3 (4.1–4.5)	135	1.08 (1.00, 1.17)
Bladder cancer	383	1.0 (0.9–1.0)	358	0.9 (0.8–1.0)	−25	0.91 (0.78, 1.05)
Non-Hodgkin’s lymphoma	846	2.5 (2.3–2.6)	877	2.4 (2.3–2.6)	31	0.99 (0.90, 1.09)
Leukemia	768	2.4 (2.2–2.5)	777	2.4 (2.2–2.5)	9	1.00 (0.90, 1.11)

Endocrine, nutritional and metabolic diseases	4,685	11.7 (11.3–12.0)	4,947	11.7 (11.4–12.0)		1.00 (0.96, 1.04)
Diabetes mellitus	3,981	9.9 (9.6–10.2)	4,136	9.8 (9.5–10.1)	155	0.99 (0.94, 1.03)

Mental and behavioral disorders and nervous system diseases	10,440	24.0 (23.5–24.5)	10,562	22.9 (22.4–23.3)		0.95 (0.93, 0.98)
Dementia	7,243	15.4 (15.0–15.7)	7,312	14.4 (14.1–14.8)	69	0.94 (0.91, 0.97)
Alcoholism	91	0.3 (0.3–0.4)	120	0.4 (0.4–0.5)	29	1.30 (0.98, 1.71)
Parkinson’s disease	1,927	4.7 (4.5–4.9)	1,928	4.5 (4.3–4.7)	1	0.95 (0.89, 1.02)

Diseases of the circulatory system	31,580	75.3 (74.4–76.1)	32,628	73.3 (72.5–74.1)		0.97 (0.96, 0.99)
Hypertensive diseases	3,836	8.4 (8.1–8.6)	4,103	8.4 (8.1–8.7)	267	1.00 (0.96, 1.05)
Ischemic heart diseases	6,003	14.3 (14.0–14.7)	6,097	13.8 (13.4–14.2)	94	0.96 (0.93, 1.00)
Heart failure	4,490	9.9 (9.6–10.2)	4,836	10.0 (9.7–10.3)	346	1.01 (0.97, 1.05)
Cerebrovascular diseases	10,960	27.2 (26.7–27.7)	11,230	26.3 (25.8–26.8)	270	0.97 (0.94, 0.99)
Hemorrhagic stroke	3,381	9.5 (9.1–9.8)	3,558	9.5 (9.2–9.8)	177	1.00 (0.95, 1.05)
Ischemic stroke	3,668	8.4 (8.2–8.7)	3,800	8.2 (7.9–8.4)	132	0.97 (0.93, 1.02)
Other stroke	3,911	9.3 (9.0–9.6)	3,872	8.6 (8.3–8.9)	−39	0.93 (0.89, 0.97)

Diseases of the respiratory system	16,142	36.7 (36.1–37.3)	15,697	33.7 (33.2–34.3)		0.92 (0.90, 0.94)
Pneumonia	11,011	24.7 (24.3–25.2)	10,172	21.6 (21.2–22.0)	−839	0.87 (0.85, 0.90)
Chronic lower respiratory diseases	2,121	4.8 (4.6–5.0)	1,990	4.2 (4.0–4.4)	−131	0.88 (0.82, 0.93)
Pneumonitis due to solids and liquids	1,106	2.5 (2.4–2.7)	1,476	3.2 (3.0–3.3)	370	1.26 (1.16, 1.37)
Interstitial pulmonary diseases	633	1.7 (1.5–1.8)	621	1.5 (1.4–1.7)	−12	0.93 (0.83, 1.04)

Diseases of the digestive system	4,647	12.3 (11.9–12.7)	4,948	12.5 (12.1–12.9)		1.01 (0.97, 1.06)
Liver diseases	1,724	5.3 (5.0–5.6)	1,787	5.4 (5.1–5.7)	63	1.02 (0.95, 1.09)
Alcoholic liver disease	518	1.9 (1.8–2.1)	587	2.2 (2.0–2.4)	69	1.12 (0.99, 1.26)
Liver cirrhosis	885	2.5 (2.3–2.7)	869	2.3 (2.2–2.5)	−16	0.94 (0.85, 1.03)

Diseases of the genitourinary system	4,853	11.7 (11.4–12.1)	5,230	11.9 (11.6–12.3)		1.02 (0.98, 1.06)
Renal failure	3,046	7.5 (7.3–7.8)	3,266	7.6 (7.4–7.9)	220	1.01 (0.96, 1.07)

Symptoms, signs and abnormal clinical and laboratory findings	15,634	35.2 (34.6–35.8)	17,666	37.0 (36.4–37.5)		1.05 (1.03, 1.07)
Senility	9,312	19.3 (18.9–19.7)	10,673	20.4 (20.0–20.8)	1,361	1.06 (1.03, 1.09)
Other ill-defined and unspecified causes of mortality	3,158	8.5 (8.2–8.8)	3,541	8.8 (8.5–9.1)	383	1.04 (0.99, 1.09)

External causes of morbidity and mortality	8,386	27.1 (26.5–27.7)	8,410	26.6 (26.0–27.2)		0.98 (0.95, 1.01)
Transport accidents	1,059	3.3 (3.1–3.5)	992	3.0 (2.8–3.2)	−67	0.90 (0.83, 0.99)
Falls	737	2.0 (1.8–2.1)	737	1.9 (1.7–2.0)	0	0.93 (0.84, 1.04)
Intentional self-harm	4,069	15.1 (14.6–15.5)	4,102	15.2 (14.7–15.6)	33	1.01 (0.96, 1.05)

Sub-specific causes						
Alcohol-specific disorders and poisonings	641	2.4 (2.2–2.6)	732	2.7 (2.5–2.9)	91	1.13 (1.02, 1.26)
Smoking-related causes (+35 yr)	63,876	159.6 (158.3–160.8)	64,129	152.4 (151.2–153.7)	253	0.96 (0.94, 0.97)
Avoidable causes (0–74 yr)	22,888	82.5 (81.4–83.5)	23,034	81.1 (80.0–82.1)	146	0.98 (0.97, 1.00)
Amenable causes	11,743	41.6 (40.9–42.4)	11,844	40.7 (40.0–41.5)	101	0.98 (0.95, 1.00)
Preventable causes	16,066	58.0 (57.1–58.9)	16,106	56.9 (56.0–57.8)	40	0.98 (0.96, 1.00)

COVID-19, coronavirus disease 2019; RR, rate ratio; CI, confidence interval.

**Table 4:** Age-specific mortality rates and differences between 2019 and 2020 by specific causes among Koreans

Gender	2019		2020		Between 2019 and 2020	
		
	No. of deaths	Age-specific mortality rates (per 100,000)	No. of deaths	Age-specific mortality rates (per 100,000)	No. of difference	RR (95% CI)
Total						
0–4	1,055	55 (52–59)	859	49 (46–52)	−196	0.88 (0.81, 0.97)
5–9	178	8 (7–9)	142	6 (5–7)	−36	0.80 (0.64, 1.00)
10–14	184	8 (7–9)	203	9 (8–10)	19	1.10 (0.90, 1.34)
15–19	610	22 (21–24)	563	22 (20–24)	−47	0.99 (0.88, 1.11)
20–24	1,126	34 (32–36)	1,118	34 (32–36)	−8	1.02 (0.94, 1.11)
25–29	1,435	42 (40–44)	1,588	45 (43–48)	153	1.08 (1.01, 1.16)
30–34	1,788	57 (54–59)	1,752	56 (53–59)	−36	0.99 (0.92, 1.05)
35–39	3,117	79 (76–82)	3,007	79 (77–82)	−110	1.01 (0.96, 1.06)
40–44	4,219	109 (106–113)	4,155	108 (104–111)	−64	0.98 (0.94, 1.03)
45–49	7,701	172 (168–176)	7,419	169 (166–173)	−282	0.98 (0.95, 1.02)
50–54	11,096	261 (256–265)	10,802	249 (245–254)	−294	0.96 (0.93, 0.98)
55–59	16,195	380 (374–386)	15,588	371 (365–376)	−607	0.97 (0.95, 1.00)
60–64	19,261	535 (527–542)	19,894	523 (516–530)	633	0.98 (0.96, 1.00)
65–69	20,181	826 (814–837)	21,201	804 (794–815)	1,020	0.97 (0.96, 0.99)
70–74	26,083	1,371 (1,355–1,388)	26,466	1,323 (1,307–1,339)	383	0.96 (0.95, 0.98)
75–79	42,208	2,633 (2,608–2,658)	41,835	2,610 (2,585–2,635)	−373	0.99 (0.98, 1.01)
80–84	53,140	5,031 (4,989–5,074)	55,154	4,965 (4,923–5,006)	2,014	0.99 (0.98, 1.00)
85+	85,533	11,994 (11,914–12,074)	93,202	11,876 (11,800–11,952)	7,669	0.99 (0.98, 1.00)

Men						
0–4	587	60 (55–65)	493	55 (50–59)	−94	0.91 (0.81, 1.03)
5–9	108	9 (7–11)	80	7 (5–8)	−28	0.75 (0.56, 1.00)
10–14	108	9 (7–11)	121	10 (8–12)	13	1.12 (0.86, 1.45)
15–19	374	26 (24–29)	365	28 (25–30)	−9	1.04 (0.90, 1.21)
20–24	682	39 (36–42)	650	38 (35–41)	−32	0.98 (0.88, 1.09)
25–29	938	52 (49–55)	1,010	55 (51–58)	72	1.05 (0.96, 1.15)
30–34	1,132	70 (66–74)	1,108	68 (64–72)	−24	0.98 (0.90, 1.07)
35–39	1,990	99 (94–103)	1,860	96 (92–101)	−130	0.98 (0.92, 1.04)
40–44	2,755	141 (135–146)	2,702	138 (133–143)	−53	0.98 (0.93, 1.03)
45–49	5,328	234 (228–241)	5,035	226 (220–233)	−293	0.97 (0.93, 1.00)
50–54	8,096	377 (369–385)	7,728	354 (346–362)	−368	0.94 (0.91, 0.97)
55–59	11,942	559 (549–569)	11,443	541 (531–551)	−499	0.97 (0.94, 0.99)
60–64	14,219	800 (787–813)	14,534	774 (761–786)	315	0.97 (0.95, 0.99)
65–69	14,417	1,224 (1,204–1,244)	15,240	1,198 (1,179–1,217)	823	0.98 (0.96, 1.00)
70–74	17,633	1,995 (1,966–2,025)	18,128	1,940 (1,912–1,969)	495	0.97 (0.95, 0.99)
75–79	25,443	3,765 (3,718–3,811)	25,521	3,742 (3,696–3,787)	78	0.99 (0.98, 1.01)
80–84	27,070	6,999 (6,916–7,082)	28,418	6,875 (6,795–6,955)	1,348	0.98 (0.97, 1.00)
85+	27,500	14,439 (14,269–14,610)	30,727	14,332 (14,171–14,492)	3,227	0.99 (0.98, 1.01)

Women						
0–4	468	50 (46–55)	366	43 (38–47)	−102	0.85 (0.74, 0.97)
5–9	70	6 (5–8)	62	6 (4–7)	−8	0.89 (0.63, 1.25)
10–14	76	7 (5–8)	82	7 (6–9)	6	1.07 (0.79, 1.47)
15–19	236	18 (16–20)	198	16 (14–18)	−38	0.89 (0.74, 1.08)
20–24	444	28 (25–30)	468	30 (27–33)	24	1.08 (0.94, 1.22)
25–29	497	31 (28–33)	578	35 (32–38)	81	1.14 (1.01, 1.29)
30–34	656	43 (40–46)	644	43 (39–46)	−12	0.99 (0.89, 1.11)
35–39	1,127	58 (55–61)	1,147	62 (58–66)	20	1.07 (0.98, 1.16)
40–44	1,464	77 (73–81)	1,453	76 (73–80)	−11	0.99 (0.92, 1.06)
45–49	2,373	108 (103–112)	2,384	111 (106–115)	11	1.03 (0.97, 1.09)
50–54	3,000	142 (137–147)	3,074	143 (138–148)	74	1.01 (0.96, 1.06)
55–59	4,253	200 (194–206)	4,145	198 (192–204)	−108	0.99 (0.95, 1.03)
60–64	5,042	277 (269–284)	5,360	278 (271–286)	318	1.01 (0.97, 1.05)
65–69	5,764	455 (444–467)	5,961	437 (426–448)	197	0.96 (0.93, 1.00)
70–74	8,450	830 (812–847)	8,338	782 (765–799)	−112	0.94 (0.91, 0.97)
75–79	16,765	1,808 (1,780–1,835)	16,314	1,772 (1,745–1,799)	−451	0.98 (0.96, 1.00)
80–84	26,070	3,895 (3,847–3,942)	26,736	3,833 (3,787–3,879)	666	0.98 (0.97, 1.00)
85+	58,033	11,103 (11,013–11,193)	62,475	10,953 (10,867–11,039)	4,442	0.99 (0.98, 1.00)

RR, rate ratio; CI, confidence interval.
