# Supplementary material for: Cause-specific mortality in Korea during the first year of the COVID-19 pandemic
Source: Epidemiol Health. 2022 Nov 23;44:e2022110. doi: 10.4178/epih.e2022110 (PMC10106553; doi:10.4178/epih.e2022110)
Supplement: Supplementary file 1 [file epih-44-e2022110-Supplementary-1.docx]

Supplementary Material 1. Age-standardized mortality rates from 1998 to 2020 among Korean

| 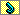 | No. of population | No. of deaths | Age-standardized mortality rates (per 100,000) |
| --- | --- | --- | --- |
| **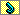Men and women combined** | |  |  |
| 1998 | 46837620 | 245825 | 993.5 (989.4 - 997.6) |
| 1999 | 47163425 | 247734 | 972.4 (968.4 - 976.4) |
| 2000 | 47534117 | 248740 | 945.7 (941.8 - 949.6) |
| 2001 | 47877049 | 243813 | 896.7 (893.0 - 900.5) |
| 2002 | 48125745 | 247524 | 880.7 (877.1 - 884.3) |
| 2003 | 48308386 | 246463 | 838.0 (834.5 - 841.4) |
| 2004 | 48485314 | 246220 | 807.8 (804.5 - 811.1) |
| 2005 | 48683040 | 245874 | 775.5 (772.3 - 778.6) |
| 2006 | 48887027 | 244162 | 741.3 (738.3 - 744.4) |
| 2007 | 49130354 | 246482 | 712.8 (709.9 - 715.7) |
| 2008 | 49404648 | 246113 | 675.8 (673.1 - 678.5) |
| 2009 | 49656756 | 246942 | 644.0 (641.5 - 646.6) |
| 2010 | 49879812 | 255405 | 637.0 (634.5 - 639.5) |
| 2011 | 50111476 | 257396 | 614.3 (611.9 - 616.7) |
| 2012 | 50345325 | 267221 | 609.2 (606.9 - 611.5) |
| 2013 | 50558952 | 266257 | 577.0 (574.8 - 579.2) |
| 2014 | 50763158 | 267692 | 551.9 (549.8 - 554.0) |
| 2015 | 50951719 | 275895 | 541.5 (539.5 - 543.5) |
| 2016 | 51112972 | 280827 | 525.7 (523.7 - 527.6) |
| 2017 | 51230704 | 285534 | 507.4 (505.6 - 509.3) |
| 2018 | 51301008 | 298820 | 504.6 (502.8 - 506.4) |
| 2019 | 51337424 | 295110 | 475.6 (473.8 - 477.3) |
| 2020 | 51349259 | 304948 | 467.4 (465.7 - 469.1) |
| **Men** |  |  |  |
| 1998 | 23519754 | 137879 | 1,365.7 (1,357.3 - 1,374.0) |
| 1999 | 23679249 | 137637 | 1,326.2 (1,318.2 - 1,334.3) |
| 2000 | 23863276 | 137789 | 1,284.6 (1,276.8 - 1,292.4) |
| 2001 | 24032276 | 135218 | 1,219.0 (1,211.6 - 1,226.4) |
| 2002 | 24151328 | 135846 | 1,190.7 (1,183.5 - 1,197.9) |
| 2003 | 24235168 | 135885 | 1,134.1 (1,127.3 - 1,140.9) |
| 2004 | 24316613 | 136235 | 1,097.8 (1,091.3 - 1,104.4) |
| 2005 | 24409659 | 135317 | 1,054.4 (1,048.1 - 1,060.6) |
| 2006 | 24506619 | 134631 | 1,011.4 (1,005.5 - 1,017.4) |
| 2007 | 24624127 | 135664 | 976.3 (970.6 - 982.0) |
| 2008 | 24757073 | 136932 | 938.2 (932.8 - 943.6) |
| 2009 | 24876418 | 137735 | 893.5 (888.4 - 898.6) |
| 2010 | 24977164 | 142358 | 886.0 (881.1 - 890.9) |
| 2011 | 25081788 | 143250 | 855.1 (850.4 - 859.8) |
| 2012 | 25187494 | 147372 | 845.0 (840.5 - 849.6) |
| 2013 | 25282928 | 146599 | 797.6 (793.3 - 801.9) |
| 2014 | 25374486 | 147321 | 762.7 (758.7 - 766.8) |
| 2015 | 25458058 | 150449 | 745.0 (741.2 - 748.9) |
| 2016 | 25527815 | 152529 | 720.4 (716.7 - 724.1) |
| 2017 | 25576752 | 154328 | 693.0 (689.4 - 696.5) |
| 2018 | 25602027 | 161187 | 687.0 (683.6 - 690.4) |
| 2019 | 25609342 | 160322 | 649.0 (645.8 - 652.2) |
| 2020 | 25606081 | 165163 | 636.3 (633.2 - 639.4) |
| **Women** |  |  |  |
| 1998 | 23317867 | 107946 | 738.6 (734.1 - 743.1) |
| 1999 | 23484176 | 110097 | 728.6 (724.2 - 733.0) |
| 2000 | 23670842 | 110951 | 710.7 (706.5 - 714.9) |
| 2001 | 23844773 | 108595 | 672.0 (667.9 - 676.0) |
| 2002 | 23974417 | 111678 | 665.0 (661.1 - 669.0) |
| 2003 | 24073218 | 110578 | 630.3 (626.5 - 634.0) |
| 2004 | 24168701 | 109985 | 602.7 (599.1 - 606.3) |
| 2005 | 24273381 | 110557 | 580.4 (577.0 - 583.9) |
| 2006 | 24380408 | 109531 | 551.4 (548.2 - 554.7) |
| 2007 | 24506227 | 110818 | 529.3 (526.1 - 532.4) |
| 2008 | 24647575 | 109181 | 493.6 (490.7 - 496.6) |
| 2009 | 24780338 | 109207 | 468.7 (465.9 - 471.4) |
| 2010 | 24902648 | 113047 | 461.2 (458.5 - 463.9) |
| 2011 | 25029688 | 114146 | 443.3 (440.8 - 445.9) |
| 2012 | 25157831 | 119849 | 441.1 (438.6 - 443.6) |
| 2013 | 25276024 | 119658 | 418.0 (415.6 - 420.4) |
| 2014 | 25388672 | 120371 | 398.3 (396.0 - 400.5) |
| 2015 | 25493662 | 125446 | 392.4 (390.2 - 394.6) |
| 2016 | 25585157 | 128298 | 381.7 (379.6 - 383.8) |
| 2017 | 25653952 | 131206 | 368.1 (366.1 - 370.1) |
| 2018 | 25698981 | 137633 | 366.3 (364.3 - 368.3) |
| 2019 | 25728082 | 134788 | 342.7 (340.8 - 344.6) |
| 2020 | 25743179 | 139785 | 337.2 (335.4 - 339.1) |
