# Supplementary material for: Cause-specific mortality in Korea during the first year of the COVID-19 pandemic
Source: Epidemiol Health. 2022 Nov 23;44:e2022110. doi: 10.4178/epih.e2022110 (PMC10106553; doi:10.4178/epih.e2022110)
Supplement: Supplementary file 2 [file epih-44-e2022110-Supplementary-2.docx]

Supplementary Material 2. The list of causes of death considered amenable and preventable (age threshold: 0-74)

| **Condition group and causes of deaths** | **ICD-10 codes** | **Preventable** | **Treatable** |
| --- | --- | --- | --- |
| **Infectious diseases** | | | |
| Intestinal diseases | A00-A09 | x |  |
| Diphtheria, Tetanus, Poliomyelitis | A35, A36, A80 | x |  |
| Whooping cough | A37 | x |  |
| Meningococcal infection | A39 | x |  |
| Sepsis due to streptococcus pneumonia and sepsis due to Haemophilus influenzae | A40.3, A41.3 | x |  |
| Haemophilus influenza infections | A49.2 | x |  |
| Sexually transmitted infections (except HIV/AIDS) | A50-A60, A63, A64 | x |  |
| Varicella | B01 | x |  |
| Measles | B05 | x |  |
| Rubella | B06 | x |  |
| Viral Hepatitis | B15-B19 | x |  |
| HIV/AIDS | B20-B24 | x |  |
| Malaria | B50-B54 | x |  |
| Haemophilus and pneumococcal meningitis | G00.0, G00.1 | x |  |
| Tuberculosis | A15-A19, B90, J65 | x | x |
| Scarlet fever | A38 |  | x |
| Sepsis | A40 (excl. A40.3), A41 (excl. A41.3) |  | x |
| Cellulitis | A46, L03 |  | x |
| Legionnaires disease | A48.1 |  | x |
| Streptococcal and enterococci infection | A49.1 |  | x |
| Other meningitis | G00.2, G00.3, G00.8, G00.9 |  | x |
| Meningitis due to other and unspecified causes | G03 |  | x |
| COVID-19 | U07.1- U07.2 | x |  |
| **Cancer** | | | |
| Lip, oral cavity and pharynx cancer | C00-C14 | x |  |
| Oesophageal cancer | C15 | x |  |
| Stomach cancer | C16 | x |  |
| Liver cancer | C22 | x |  |
| Lung cancer | C33-C34 | x |  |
| Mesothelioma | C45 | x |  |
| Skin (melanoma) cancer | C43 | x |  |
| Bladder cancer | C67 | x |  |
| Cervical cancer | C53 | x | x |
| Colorectal cancer | C18-C21 |  | x |
| Breast cancer (female only) | C50 |  | x |
| Uterus cancer | C54, C55 |  | x |
| Testicular cancer | C62 |  | x |
| Thyroid cancer | C73 |  | x |
| Hodgkin's disease | C81 |  | x |
| Lymphoid leukaemia | C91.0, C91.1 |  | x |
| Benign neoplasm | D10-D36 |  | x |
| **Endocrine and metabolic diseases** | | | |
| Nutritional deficiency anaemia | D50-D53 | x |  |
| Diabetes mellitus | E10-E14 | x | x |
| Thyroid disorders | E00-E07 |  | x |
| Adrenal disorders | E24-E25 (except E24.4), E27 |  | x |
| **Diseases of the nervous system** | | | |
| Epilepsy | G40, G41 |  | x |
| **Diseases of the circulatory system** | | | |
| Aortic aneurysm | I71 | x | x |
| Hypertensive diseases | I10-I13, I15 | x | x |
| Ischaemic heart diseases | I20-I25 | x | x |
| Cerebrovascular diseases | I60-I69 | x | x |
| Other atherosclerosis | I70, I73.9 | x | x |
| Rheumatic and other heart disease | I00-I09 |  | x |
| Venous thromboembolism | I26, I80, I82.9 |  | x |
| **Diseases of the respiratory system** | | | |
| Influenza | J09-J11 | x |  |
| Pneumonia due to Streptococcus pneumonia or Haemophilus influenza | J13-J14 | x |  |
| Chronic lower respiratory diseases | J40-J44 | x |  |
| Lung diseases due to external agents | J60-J64, J66-J70, J82, J92 | x |  |
| Upper respiratory infections | J00-J06, J30-J39 |  | x |
| Pneumonia, not elsewhere classified or organism unspecified | J12, J15, J16- J18 |  | x |
| Acute lower respiratory infections | J20-J22 |  | x |
| Asthma and bronchiectasis | J45-J47 |  | x |
| Adult respiratory distress syndrome | J80 |  | x |
| Pulmonary oedema | J81 |  | x |
| Abscess of lung and mediastinum pyothorax | J85, J86 |  | x |
| Other pleural disorders | J90, J93, J94 |  | x |
| **Diseases of the digestive system** | | | |
| Gastric and duodenal ulcer | K25-K28 |  | x |
| Appendicitis | K35-K38 |  | x |
| Abdominal hernia | K40-K46 |  | x |
| Cholelithiasis and cholecystitis | K80-K81 |  | x |
| Other diseases of gallbladder or biliary tract | K82-K83 |  | x |
| Acute pancreatitis | K85.0,1,3,8,9 |  | x |
| Other diseases of pancreas | K86.1,2,3,8,9 |  | x |
| **Diseases of the genitourinary system** | | | |
| Nephritis and nephrosis | N00-N07 |  | x |
| Obstructive uropathy | N13, N20-N21, N35 |  | x |
| Renal failure | N17-N19 |  | x |
| Renal colic | N23 |  | x |
| Disorders resulting from renal tubular dysfunction | N25 |  | x |
| Unspecified contracted kidney, small kidney of unknown cause | N26-N27 |  | x |
| Inflammatory diseases of genitourinary system | N34.1, N70- N73, N75.0, N75.1, N76.4,6 |  | x |
| Prostatic hyperplasia | N40 |  | x |
| **Pregnancy, childbirth, and perinatal period** | | | |
| Tetanus neonatorum | A33 | x |  |
| Obstetrical tetanus | A34 | x |  |
| Pregnancy, childbirth and the puerperium | O00-O99 |  | x |
| Certain conditions originating in the perinatal period | P00-P96 |  | x |
| **Congenital malformations** | | | |
| Certain congenital malformations (neural tube defects) | Q00, Q01, Q05 | x |  |
| Congenital malformations of the circulatory system (heart defects) | Q20-Q28 |  | x |
| **Adverse effects of medical and surgical care** | | | |
| Drugs, medicaments and biological substances causing adverse effects in therapeutic use | Y40-Y59 |  | x |
| Misadventures to patients during surgical and medical care | Y60-Y69, Y83-Y84 |  | x |
| Medical devices associated with adverse incidents in diagnostic and therapeutic use | Y70–Y82 |  | x |
| **Injuries** | | | |
| Transport Accidents | V01-V99 | x |  |
| Accidental Injuries | W00-X39, X46-X59 | x |  |
| Intentional self-harm | X66-X84 | x |  |
| Event of undetermined intent | Y16-Y34 | x |  |
| Assault | X86-Y09 | x |  |
| **Alcohol-related and drug-related deaths** | | | |
| Alcohol- specific disorders and poisonings | E24.4, F10, G31.2, G62.1, G72.1, I42.6, K29.2, K70, K85.2, K86.0, Q86.0, R78.0, X45, X65, Y15 | x |  |
| Other alcohol-related disorders | K73, K74.0-K74.2, K74.6 | x |  |
| Drug disorders and poisonings | F11-F16, F18-F19, X40- X44, X85, Y10-Y14 | x |  |
| Intentional self-poisoning by drugs | X60-X64 | x |  |
