# Supplementary material for: Cause-specific mortality in Korea during the first year of the COVID-19 pandemic
Source: Epidemiol Health. 2022 Nov 23;44:e2022110. doi: 10.4178/epih.e2022110 (PMC10106553; doi:10.4178/epih.e2022110)
Supplement: Supplementary file 3 [file epih-44-e2022110-Supplementary-3.docx]

Supplementary Material 3. Age-standardized death rates by specific causes of death in 2015-2019 (combined), 2018, 2019, and 2020 among Korean men and women

| **Causes** | 2015-2019 combined | |  | 2018 | | |  | 2019 | | |  | 2020 | | |  | Between 2019 and 2020 | | |
| --- | --- | --- | --- | --- | --- | --- | --- | --- | --- | --- | --- | --- | --- | --- | --- | --- | --- | --- |
|  | No of deaths | Age-standardized mortality rates  (per 100,000) |  | No of deaths | Age-standardized mortality rates  (per 100,000) | % |  | No of deaths | Age-standardized mortality rates  (per 100,000) | % |  | No of deaths | Age-standardized mortality rates  (per 100,000) | % |  | Number difference | Rate difference | Rate ratio |
| **Certain infectious and parasitic diseases(A00-B99, U07.1, U07.2, U08-U10)** | **40665** | **14.3 (14.1 - 14.4)** |  | **8746** | **14.5 (14.2 - 14.8)** | **2.9** |  | **8692** | **13.7 (13.4 - 14.0)** | **2.9** |  | **10419** | **15.5 (15.2 - 15.8)** | **3.3** |  |  | **1.8 (1.4 to 2.2)** | **1.13 (1.10 to 1.16)** |
| Tuberculosis (A15-A19) | 9621 | 3.4 (3.3 - 3.4) |  | 1800 | 3.0 (2.9 - 3.1) | 0.6 |  | 1610 | 2.5 (2.4 - 2.7) | 0.5 |  | 1356 | 2.0 (1.9 - 2.1) | 0.4 |  | -254 | -0.5 (-0.7 to -0.3) | 0.81 (0.75 to 0.87) |
| Sepsis (A40-A41) | 20203 | 7.0 (6.9 - 7.1) |  | 4665 | 7.7 (7.5 - 7.9) | 1.5 |  | 4903 | 7.6 (7.4 - 7.8) | 1.6 |  | 6086 | 8.9 (8.7 - 9.1) | 1.9 |  | 1183 | 1.3 (1.0 to 1.6) | 1.17 (1.13 to 1.22) |
| Covid19 (U07.1, U07.2, U08-U10) | 0 | 0 |  | 0 | 0 | 0.0 |  | 0 | 0 | 0.0 |  | 950 | 1.4 (1.3 - 1.5) | 0.3 |  | 950 | - | - |
| **Malignant neoplasms(C00-C97)** | **394268** | **141.7 (141.3 - 142.2)** |  | **79153** | **136.1 (135.2 - 137.1)** | **27.0** |  | **81203** | **134.0 (133.1 - 134.9)** | **28.2** |  | **82204** | **130.1 (129.2 - 131.0)** | **27.8** |  |  | **-3.9 (-5.2 to -2.6)** | **0.97 (0.96 to 0.98)** |
| Oesophageal cancer (C15) | 7467 | 2.7 (2.6 - 2.7) |  | 1435 | 2.5 (2.3 - 2.6) | 0.5 |  | 1554 | 2.6 (2.4 - 2.7) | 0.5 |  | 1564 | 2.5 (2.4 - 2.6) | 0.5 |  | 10 | -0.1 (-0.3 to 0.1) | 0.96 (0.90 to 1.03) |
| Stomach cancer (C16) | 40194 | 14.4 (14.3 - 14.6) |  | 7746 | 13.3 (13.0 - 13.6) | 2.6 |  | 7624 | 12.5 (12.3 - 12.8) | 2.6 |  | 7510 | 11.9 (11.6 - 12.1) | 2.5 |  | -114 | -0.7 (-1.1 to -0.3) | 0.95 (0.92 to 0.98) |
| Colorectal cancer (C18-C21) | 43330 | 15.5 (15.3 - 15.6) |  | 8786 | 14.9 (14.6 - 15.3) | 3.0 |  | 8966 | 14.6 (14.3 - 14.9) | 3.1 |  | 8944 | 13.9 (13.6 - 14.2) | 3.0 |  | -22 | -0.7 (-1.1 to -0.3) | 0.95 (0.92 to 0.98) |
| Liver cancer (C22) | 54230 | 19.7 (19.5 - 19.8) |  | 10611 | 18.5 (18.1 - 18.8) | 3.7 |  | 10586 | 17.8 (17.4 - 18.1) | 3.7 |  | 10565 | 17.0 (16.7 - 17.3) | 3.6 |  | -21 | -0.8 (-1.2 to -0.3) | 0.96 (0.93 to 0.98) |
| Gallbladder cancer (C23) | 8888 | 3.2 (3.1 - 3.2) |  | 1738 | 3.0 (2.8 - 3.1) | 0.6 |  | 1906 | 3.1 (2.9 - 3.2) | 0.6 |  | 1890 | 2.9 (2.8 - 3.0) | 0.6 |  | -16 | -0.2 (-0.4 to 0.0) | 0.95 (0.89 to 1.01) |
| Biliary tract cancer (C24) | 14307 | 5.1 (5.0 - 5.2) |  | 3133 | 5.3 (5.1 - 5.5) | 1.0 |  | 3082 | 5.0 (4.8 - 5.1) | 1.0 |  | 3302 | 5.1 (4.9 - 5.2) | 1.1 |  | 220 | 0.1 (-0.1 to 0.3) | 1.02 (0.97 to 1.07) |
| Pancreatic cancer (C25) | 29267 | 10.5 (10.4 - 10.6) |  | 6036 | 10.3 (10.1 - 10.6) | 2.1 |  | 6396 | 10.5 (10.3 - 10.8) | 2.2 |  | 6775 | 10.7 (10.4 - 10.9) | 2.3 |  | 379 | 0.2 (-0.2 to 0.5) | 1.01 (0.98 to 1.05) |
| Lung cancer (C33-C34) | 89768 | 32.1 (31.9 - 32.3) |  | 17852 | 30.4 (30.0 - 30.9) | 6.0 |  | 18574 | 30.3 (29.8 - 30.7) | 6.4 |  | 18673 | 29.1 (28.7 - 29.5) | 6.2 |  | 99 | -1.1 (-1.8 to -0.5) | 0.96 (0.94 to 0.98) |
| Breast cancer (C50), Female | 12373 | 4.6 (4.5 - 4.7) |  | 2460 | 4.5 (4.3 - 4.6) | 0.9 |  | 2622 | 4.6 (4.5 - 4.8) | 1.0 |  | 2725 | 4.7 (4.5 - 4.9) | 1.0 |  | 103 | 0.1 (-0.2 to 0.3) | 1.02 (0.96 to 1.07) |
| Cervical cancer (C53) | 4475 | 1.6 (1.6 - 1.7) |  | 845 | 1.5 (1.4 - 1.6) | 0.3 |  | 898 | 1.6 (1.4 - 1.7) | 0.3 |  | 810 | 1.4 (1.3 - 1.5) | 0.3 |  | -88 | -0.2 (-0.3 to 0.0) | 0.88 (0.80 to 0.97) |
| Uterus cancer (C54-C55) | 2087 | 0.8 (0.7 - 0.8) |  | 423 | 0.7 (0.7 - 0.8) | 0.1 |  | 443 | 0.8 (0.7 - 0.8) | 0.2 |  | 474 | 0.8 (0.7 - 0.9) | 0.2 |  | 31 | 0.0 (-0.1 to 0.1) | 1.03 (0.91 to 1.18) |
| Ovarian cancer (C56) | 5885 | 2.2 (2.1 - 2.2) |  | 1243 | 2.2 (2.1 - 2.3) | 0.4 |  | 1234 | 2.1 (2.0 - 2.2) | 0.4 |  | 1369 | 2.3 (2.2 - 2.4) | 0.5 |  | 135 | 0.2 (0.0 to 0.3) | 1.08 (1.00 to 1.16) |
| Prostate cancer (C61) | 9308 | 3.3 (3.2 - 3.3) |  | 1995 | 3.3 (3.2 - 3.4) | 0.7 |  | 2047 | 3.2 (3.1 - 3.4) | 0.7 |  | 2194 | 3.3 (3.1 - 3.4) | 0.7 |  | 147 | 0.1 (-0.1 to 0.2) | 1.02 (0.96 to 1.08) |
| Kidney cancer (C64) | 4956 | 1.8 (1.7 - 1.8) |  | 989 | 1.7 (1.6 - 1.8) | 0.3 |  | 983 | 1.6 (1.5 - 1.7) | 0.3 |  | 1076 | 1.7 (1.6 - 1.8) | 0.4 |  | 93 | 0.1 (-0.1 to 0.2) | 1.06 (0.97 to 1.15) |
| Bladder cancer (C67) | 7076 | 2.5 (2.4 - 2.5) |  | 1400 | 2.3 (2.2 - 2.5) | 0.5 |  | 1550 | 2.4 (2.3 - 2.6) | 0.5 |  | 1593 | 2.4 (2.3 - 2.5) | 0.5 |  | 43 | -0.1 (-0.2 to 0.1) | 0.98 (0.91 to 1.05) |
| Brain cancer (C70-72) | 6683 | 2.5 (2.4 - 2.5) |  | 1355 | 2.4 (2.3 - 2.6) | 0.5 |  | 1416 | 2.5 (2.3 - 2.6) | 0.5 |  | 1437 | 2.4 (2.3 - 2.6) | 0.5 |  | 21 | 0.0 (-0.2 to 0.1) | 0.98 (0.91 to 1.06) |
| Non-Hodgkin's lymphoma (C82-C86) | 9451 | 3.4 (3.3 - 3.5) |  | 2033 | 3.5 (3.4 - 3.7) | 0.7 |  | 2015 | 3.3 (3.2 - 3.5) | 0.7 |  | 2069 | 3.3 (3.2 - 3.4) | 0.7 |  | 54 | 0.0 (-0.3 to 0.2) | 0.99 (0.93 to 1.05) |
| Multiple myeloma (C90) | 4777 | 1.7 (1.7 - 1.8) |  | 953 | 1.6 (1.5 - 1.7) | 0.3 |  | 961 | 1.6 (1.5 - 1.7) | 0.3 |  | 976 | 1.5 (1.4 - 1.6) | 0.3 |  | 15 | 0.0 (-0.2 to 0.1) | 0.98 (0.89 to 1.07) |
| Leukaemia (C91-C95) | 9155 | 3.4 (3.3 - 3.4) |  | 1848 | 3.3 (3.1 - 3.4) | 0.7 |  | 1911 | 3.3 (3.1 - 3.4) | 0.7 |  | 1825 | 3.0 (2.9 - 3.2) | 0.7 |  | -86 | -0.2 (-0.4 to 0.0) | 0.93 (0.87 to 0.99) |
| Other neoplasms(D00-D48) | 7653 | 2.7 (2.7 - 2.8) |  | **1594** | **2.7 (2.6 - 2.8)** | **0.5** |  | 1641 | 2.7 (2.5 - 2.8) | 0.6 |  | 1572 | 2.4 (2.3 - 2.6) | 0.5 |  | -69 | -0.2 (-0.4 to -0.1) | 0.91 (0.85 to 0.98) |
| **Endocrine, nutritional, and metabolic diseases(E00-E90)** | **53080** | **18.8 (18.6 - 18.9)** |  | **10324** | **17.3 (17.0 - 17.6)** | **3.4** |  | **9502** | **15.2 (14.9 - 15.5)** | **3.2** |  | **10052** | **15.3 (15.0 - 15.6)** | **3.3** |  |  | **0.1 (-0.3 to 0.5)** | **1.01 (0.98 to 1.03)** |
| Diabetes mellitus (E10-E14) | 46440 | 16.4 (16.2 - 16.5) |  | 8789 | 14.7 (14.4 - 15.0) | 2.9 |  | 8102 | 12.9 (12.6 - 13.2) | 2.7 |  | 8456 | 12.8 (12.5 - 13.1) | 2.7 |  | 354 | -0.1 (-0.5 to 0.3) | 0.99 (0.96 to 1.02) |
| **Mental and behavioural disorders and nervous system diseases(F00-F99, G00-G99)** | **83807** | **29.1 (28.9 - 29.3)** |  | **17439** | **28.7 (28.2 - 29.1)** | **5.7** |  | **17575** | **27.2 (26.8 - 27.6)** | **5.7** |  | **18138** | **26.5 (26.1 - 26.9)** | **5.7** |  |  | **-0.7 (-1.3 to -0.2)** | **0.97 (0.95 to 0.99)** |
| Dementia (F00-F03, G30) | 48011 | 16.3 (16.2 - 16.4) |  | 9739 | 15.5 (15.2 - 15.8) | 3.1 |  | 10357 | 15.3 (15.0 - 15.6) | 3.2 |  | 10641 | 14.6 (14.3 - 14.9) | 3.1 |  | 284 | -0.7 (-1.1 to -0.3) | 0.95 (0.93 to 0.98) |
| Alcoholism (F10) | 4469 | 1.7 (1.6 - 1.7) |  | 940 | 1.7 (1.6 - 1.8) | 0.3 |  | 916 | 1.6 (1.5 - 1.8) | 0.3 |  | 1089 | 1.9 (1.8 - 2.0) | 0.4 |  | 173 | 0.3 (0.1 to 0.4) | 1.16 (1.06 to 1.26) |
| Parkinson's disease (G20) | 18129 | 6.3 (6.2 - 6.4) |  | 3948 | 6.5 (6.3 - 6.7) | 1.3 |  | 3476 | 5.4 (5.2 - 5.6) | 1.1 |  | 3571 | 5.3 (5.1 - 5.4) | 1.1 |  | 95 | -0.1 (-0.4 to 0.1) | 0.98 (0.93 to 1.02) |
| **Diseases of the circulatory system(I00-I99)** | **304396** | **106.7 (106.3 - 107.1)** |  | **62947** | **104.4 (103.6 - 105.2)** | **20.7** |  | **60252** | **94.8 (94.0 - 95.6)** | **19.9** |  | **62196** | **92.5 (91.8 - 93.3)** | **19.8** |  |  | **-2.3 (-3.3 to -1.2)** | **0.98 (0.96 to 0.99)** |
| Hypertensive diseases (I10-I15) | 27938 | 9.6 (9.4 - 9.7) |  | 6065 | 9.7 (9.5 - 10.0) | 1.9 |  | 5631 | 8.5 (8.2 - 8.7) | 1.8 |  | 6100 | 8.6 (8.3 - 8.8) | 1.8 |  | 469 | 0.1 (-0.2 to 0.4) | 1.01 (0.98 to 1.05) |
| Ischaemic heart diseases (I20-I25) | 71834 | 25.3 (25.1 - 25.5) |  | 14500 | 24.2 (23.8 - 24.6) | 4.8 |  | 13699 | 21.8 (21.5 - 22.2) | 4.6 |  | 14056 | 21.3 (21.0 - 21.7) | 4.6 |  | 357 | -0.5 (-1.0 to 0.0) | 0.98 (0.95 to 1.00) |
| Atrial fibrillation (I48) | 5352 | 1.8 (1.8 - 1.9) |  | 1034 | 1.7 (1.6 - 1.8) | 0.3 |  | 1153 | 1.7 (1.6 - 1.8) | 0.4 |  | 1144 | 1.6 (1.5 - 1.7) | 0.3 |  | -9 | -0.1 (-0.3 to 0.0) | 0.93 (0.86 to 1.01) |
| Heart failure (I50) | 29081 | 10.0 (9.9 - 10.1) |  | 6755 | 10.9 (10.6 - 11.1) | 2.2 |  | 6758 | 10.2 (10.0 - 10.4) | 2.1 |  | 7256 | 10.2 (10.0 - 10.4) | 2.2 |  | 498 | 0.0 (-0.3 to 0.3) | 1.00 (0.97 to 1.03) |
| Cerebrovascular diseases (I60-I69) | 115141 | 40.5 (40.3 - 40.7) |  | 22940 | 38.3 (37.8 - 38.8) | 7.6 |  | 21586 | 34.2 (33.8 - 34.7) | 7.2 |  | 21860 | 32.8 (32.4 - 33.3) | 7.0 |  | 274 | -1.4 (-2.0 to -0.8) | 0.96 (0.94 to 0.98) |
| Haemorrhagic stroke (I60-I62) | 35173 | 12.7 (12.5 - 12.8) |  | 6867 | 11.9 (11.6 - 12.2) | 2.4 |  | 6942 | 11.5 (11.3 - 11.8) | 2.4 |  | 7092 | 11.3 (11.0 - 11.6) | 2.4 |  | 150 | -0.2 (-0.6 to 0.1) | 0.98 (0.95 to 1.01) |
| Ischaemic stroke (I63) | 36967 | 12.8 (12.7 - 12.9) |  | 7322 | 11.9 (11.7 - 12.2) | 2.4 |  | 7103 | 10.9 (10.7 - 11.2) | 2.3 |  | 7414 | 10.7 (10.5 - 11.0) | 2.3 |  | 311 | -0.2 (-0.6 to 0.1) | 0.98 (0.95 to 1.01) |
| Other stroke (I64-I69) | 43001 | 15.0 (14.9 - 15.1) |  | 8751 | 14.4 (14.1 - 14.7) | 2.9 |  | 7541 | 11.8 (11.5 - 12.0) | 2.5 |  | 7354 | 10.8 (10.6 - 11.1) | 2.3 |  | -187 | -0.9 (-1.3 to -0.6) | 0.92 (0.89 to 0.95) |
| Aortic aneurysm (I71) | 5276 | 1.9 (1.8 - 1.9) |  | 1110 | 1.9 (1.8 - 2.0) | 0.4 |  | 1147 | 1.9 (1.7 - 2.0) | 0.4 |  | 1106 | 1.7 (1.6 - 1.8) | 0.4 |  | -41 | -0.2 (-0.3 to 0.0) | 0.92 (0.85 to 1.00) |
| **Diseases of the respiratory system(J00-J99)** | **164272** | **56.8 (56.5 - 57.1)** |  | **37763** | **61.5 (60.9 - 62.1)** | **12.2** |  | **36655** | **56.2 (55.6 - 56.8)** | **11.8** |  | **36368** | **52.5 (52.0 - 53.1)** | **11.2** |  |  | **-3.6 (-4.4 to -2.8)** | **0.94 (0.92 to 0.95)** |
| Pneumonia (J12-J18) | 97020 | 33.4 (33.2 - 33.7) |  | 23280 | 37.7 (37.2 - 38.2) | 7.5 |  | 23168 | 35.3 (34.8 - 35.7) | 7.4 |  | 22257 | 31.9 (31.5 - 32.4) | 6.8 |  | -911 | -3.4 (-4.0 to -2.7) | 0.90 (0.89 to 0.92) |
| Chronic lower respiratory diseases (J40-J47) | 34064 | 11.8 (11.7 - 11.9) |  | 6608 | 10.8 (10.5 - 11.0) | 2.1 |  | 6176 | 9.5 (9.3 - 9.7) | 2.0 |  | 5668 | 8.2 (8.0 - 8.4) | 1.8 |  | -508 | -1.3 (-1.6 to -1.0) | 0.86 (0.83 to 0.90) |
| Pneumonitis due to solids and liquids (J69) | 11307 | 3.9 (3.8 - 4.0) |  | 2648 | 4.3 (4.1 - 4.5) | 0.9 |  | 2631 | 4.0 (3.9 - 4.2) | 0.8 |  | 3382 | 4.9 (4.7 - 5.0) | 1.0 |  | 751 | 0.8 (0.6 to 1.1) | 1.21 (1.15 to 1.27) |
| Interstitial pulmonary diseases (J84) | 8426 | 3.0 (2.9 - 3.1) |  | 1824 | 3.1 (2.9 - 3.2) | 0.6 |  | 1813 | 2.9 (2.8 - 3.1) | 0.6 |  | 1806 | 2.8 (2.6 - 2.9) | 0.6 |  | -7 | -0.2 (-0.3 to 0.0) | 0.95 (0.89 to 1.01) |
| **Diseases of the digestive system(K00-K93)** | **60154** | **21.7 (21.6 - 21.9)** |  | **12401** | **21.5 (21.1 - 21.9)** | **4.3** |  | **11963** | **20.0 (19.6 - 20.4)** | **4.2** |  | **12870** | **20.7 (20.3 - 21.0)** | **4.4** |  |  | **0.7 (0.2 to 1.2)** | **1.03 (1.01 to 1.06)** |
| Liver diseases (K70-K77) | 33796 | 12.5 (12.4 - 12.6) |  | 6858 | 12.3 (12.0 - 12.6) | 2.4 |  | 6496 | 11.4 (11.1 - 11.7) | 2.4 |  | 6979 | 11.9 (11.6 - 12.2) | 2.5 |  | 483 | 0.5 (0.1 to 0.9) | 1.05 (1.01 to 1.08) |
| Alcoholic liver disease (K70) | 18833 | 7.1 (7.0 - 7.2) |  | 3818 | 7.0 (6.8 - 7.2) | 1.4 |  | 3658 | 6.6 (6.4 - 6.8) | 1.4 |  | 3941 | 7.0 (6.8 - 7.2) | 1.5 |  | 283 | 0.4 (0.1 to 0.7) | 1.05 (1.01 to 1.10) |
| Liver cirrhosis (K74) | 11535 | 4.2 (4.1 - 4.3) |  | 2288 | 4.0 (3.8 - 4.2) | 0.8 |  | 2133 | 3.6 (3.4 - 3.8) | 0.8 |  | 2202 | 3.6 (3.5 - 3.8) | 0.8 |  | 69 | 0.0 (-0.2 to 0.2) | 1.00 (0.94 to 1.06) |
| **Diseases of the genitourinary system(N00-N99)** | **38021** | **13.3 (13.2 - 13.4)** |  | **8160** | **13.4 (13.2 - 13.7)** | **2.7** |  | **8565** | **13.3 (13.1 - 13.6)** | **2.8** |  | **9343** | **13.7 (13.5 - 14.0)** | **2.9** |  |  | **0.4 (0.0 to 0.8)** | **1.03 (1.00 to 1.06)** |
| Renal failure (N17-19) | 27202 | 9.6 (9.4 - 9.7) |  | 5657 | 9.4 (9.1 - 9.6) | 1.9 |  | 6036 | 9.5 (9.2 - 9.7) | 2.0 |  | 6589 | 9.8 (9.6 - 10.0) | 2.1 |  | 553 | 0.3 (0.0 to 0.6) | 1.03 (1.00 to 1.07) |
| **Symptoms, signs, and abnormal clinical and laboratory findings, NEC(R00-R99)** | **131556** | **45.6 (45.4 - 45.9)** |  | **28466** | **46.7 (46.2 - 47.3)** | **9.3** |  | **28176** | **43.7 (43.2 - 44.2)** | **9.2** |  | **31801** | **46.2 (45.7 - 46.8)** | **9.9** |  |  | **2.5 (1.8 to 3.2)** | **1.06 (1.04 to 1.07)** |
| Senility (R54) | 70057 | 23.7 (23.5 - 23.8) |  | 14369 | 22.6 (22.3 - 23.0) | 4.5 |  | 13522 | 19.8 (19.5 - 20.1) | 4.2 |  | 15823 | 21.4 (21.0 - 21.7) | 4.6 |  | 2301 | 1.6 (1.1 to 2.1) | 1.08 (1.06 to 1.11) |
| Other ill-defined and unspecified causes of mortality (R99) | 35654 | 12.9 (12.8 - 13.0) |  | 8067 | 14.1 (13.7 - 14.4) | 2.8 |  | 8549 | 14.3 (14.0 - 14.6) | 3.0 |  | 9143 | 14.5 (14.2 - 14.8) | 3.1 |  | 594 | 0.3 (-0.2 to 0.7) | 1.02 (0.99 to 1.05) |
| **External causes of morbidity and mortality(V01-Y98)** | **139478** | **52.0 (51.7 - 52.2)** |  | **28040** | **51.0 (50.4 - 51.6)** | **10.1** |  | **27282** | **48.5 (48.0 - 49.1)** | **10.2** |  | **26442** | **46.1 (45.5 - 46.6)** | **9.9** |  |  | **-2.5 (-3.3 to -1.7)** | **0.95 (0.93 to 0.97)** |
| Transport Accidents (V01-V99) | 24609 | 9.2 (9.1 - 9.3) |  | 4671 | 8.4 (8.2 - 8.7) | 1.7 |  | 4221 | 7.4 (7.2 - 7.7) | 1.6 |  | 3947 | 6.8 (6.6 - 7.0) | 1.5 |  | -274 | -0.6 (-1.0 to -0.3) | 0.91 (0.87 to 0.95) |
| Falls (W00-W19) | 12929 | 4.7 (4.6 - 4.8) |  | 2669 | 4.7 (4.5 - 4.8) | 0.9 |  | 2665 | 4.5 (4.3 - 4.7) | 0.9 |  | 2663 | 4.3 (4.1 - 4.4) | 0.9 |  | -2 | -0.2 (-0.5 to 0.0) | 0.95 (0.90 to 1.00) |
| Intentional self-harm (X60-X84) | 66537 | 25.3 (25.1 - 25.5) |  | 13670 | 25.7 (25.3 - 26.1) | 5.1 |  | 13799 | 25.6 (25.2 - 26.0) | 5.4 |  | 13195 | 24.4 (24.0 - 24.8) | 5.2 |  | -604 | -1.2 (-1.8 to -0.6) | 0.95 (0.93 to 0.98) |
| **Sub-specific causes groups** |  |  |  |  |  |  |  |  |  |  |  |  |  |  |  |  |  |  |
| Alcohol-specific disorders and poisonings | 24062 | 9.0 (8.9 - 9.2) |  | 4943 | 9.1 (8.8 - 9.3) | 1.8 |  | 4725 | 8.5 (8.3 - 8.8) | 1.8 |  | 5191 | 9.2 (8.9 - 9.4) | 2.0 |  | 466 | 0.6 (0.3 to 1.0) | 1.08 (1.03 to 1.12) |
| Smoking-related causes (+35 years) | 741330 | 261.7 (261.1-262.3) |  | 153047 | 256.2 (254.9 - 257.5) | 50.8 |  | 150707 | 240.2 (239.0-241.4) | 50.5 |  | 151728 | 230.0 (228.8 - 231.1) | 49.2 |  | 1021 | -17.4 (-20.2 to -14.5) | 0.96 (0.95 to 0.96) |
| Avoidable causes (0-74 years) | 424843 | 160.0 (159.6-160.5) |  | 82751 | 153.0 (152.0 - 154.1) | 30.3 |  | 81553 | 147.0 (146.0-148.0) | 30.9 |  | 81373 | 142.4 (141.4 - 143.4) | 30.5 |  | -180 | -4.8 (-6.3 to -3.3) | 0.97 (0.96 to 0.98) |
| Amenable causes | 170550 | 64.1 (63.8-64.4) |  | 33379 | 61.4 (60.8 - 62.1) | 12.2 |  | 32536 | 58.2 (57.6-58.8) | 12.2 |  | 32843 | 56.7 (56.1 - 57.3) | 12.1 |  | 307 | -1.6 (-2.5 to -0.6) | 0.97 (0.96 to 0.99) |
| Preventable causes | 346754 | 130.6 (130.2-131.0) |  | 66973 | 123.8 (122.9 - 124.8) | 24.5 |  | 65791 | 118.6 (117.7-119.5) | 24.9 |  | 65254 | 114.4 (113.5 - 115.3) | 24.5 |  | -537 | -4.4 (-5.8 to -3.1) | 0.96 (0.95 to 0.98) |
