# Supplementary material for: Cause-specific mortality in Korea during the first year of the COVID-19 pandemic
Source: Epidemiol Health. 2022 Nov 23;44:e2022110. doi: 10.4178/epih.e2022110 (PMC10106553; doi:10.4178/epih.e2022110)
Supplement: Supplementary file 4 [file epih-44-e2022110-Supplementary-4.docx]

Supplementary Material 4. Age-standardized death rates by specific causes of death in 2015-2019 (combined), 2018, 2019, and 2020 among Korean men

| **Causes** | 2015-2019 combined | |  | 2018 | | |  | 2019 | | |  | 2020 | | |  | Between 2019 and 2020 | | |
| --- | --- | --- | --- | --- | --- | --- | --- | --- | --- | --- | --- | --- | --- | --- | --- | --- | --- | --- |
|  | No of deaths | Age-standardized mortality rates  (per 100,000) |  | No of deaths | Age-standardized mortality rates  (per 100,000) | % |  | No of deaths | Age-standardized mortality rates  (per 100,000) | % |  | No of deaths | Age-standardized mortality rates  (per 100,000) | % |  | Number difference | Rate difference | Rate ratio |
| **Certain infectious and parasitic diseases(A00-B99, U07.1, U07.2, U08-U10)** | **19876** | **18.3 (18.1 - 18.6)** |  | **4209** | **18.5 (17.9 - 19.1)** |  |  | **4103** | **17.0 (16.4 - 17.5)** | **2.6** |  | **4890** | **19.2 (18.6 - 19.7)** | **3.0** |  |  | **2.2 (1.5 to 3.0)** | **1.13 (1.08 to 1.18)** |
| Tuberculosis (A15-A19) | 5943 | 5.5 (5.4 - 5.7) |  | 1120 | 4.9 (4.6 - 5.2) | 0.7 |  | 977 | 4.1 (3.8 - 4.3) | 0.6 |  | 842 | 3.3 (3.1 - 3.5) | 0.5 |  | -135 | -0.7 (-1.1 to -0.4) | 0.82 (0.74 to 0.90) |
| Sepsis (A40-A41) | 8408 | 8.0 (7.8 - 8.2) |  | 1946 | 8.8 (8.4 - 9.2) | 1.3 |  | 2013 | 8.5 (8.1 - 8.9) | 1.3 |  | 2553 | 10.1 (9.8 - 10.5) | 1.6 |  | 540 | 1.6 (1.1 to 2.2) | 1.19 (1.12 to 1.26) |
| Covid19 (U07.1, U07.2, U08-U10) | 0 | 0 |  | 0 | 0 | 0.0 |  | 0 | 0 | 0.0 |  | 483 | 1.9 (1.7 - 2.1) | 0.3 |  | 483 | - | - |
| **Malignant neoplasms(C00-C97)** | **243931** | **209.8 (208.9 - 210.6)** |  | **48898** | **200.8 (199.0 - 202.6)** |  |  | **50281** | **196.7 (195.0 - 198.5)** | **30.3** |  | **50817** | **189.6 (188.0 - 191.3)** | **29.8** |  |  | **-7.1 (-9.5 to -4.7)** | **0.96 (0.95 to 0.98)** |
| Oesophageal cancer (C15) | 6809 | 5.7 (5.6 - 5.8) |  | 1314 | 5.2 (4.9 - 5.5) | 0.8 |  | 1425 | 5.4 (5.1 - 5.7) | 0.8 |  | 1404 | 5.1 (4.8 - 5.4) | 0.8 |  | -21 | -0.3 (-0.7 to 0.1) | 0.94 (0.88 to 1.02) |
| Stomach cancer (C16) | 26030 | 22.5 (22.2 - 22.7) |  | 5083 | 21.0 (20.4 - 21.5) | 3.0 |  | 4956 | 19.5 (18.9 - 20.0) | 3.0 |  | 4807 | 17.9 (17.4 - 18.5) | 2.8 |  | -149 | -1.5 (-2.3 to -0.8) | 0.92 (0.89 to 0.96) |
| Colorectal cancer (C18-C21) | 24414 | 21.3 (21.0 - 21.6) |  | 4923 | 20.5 (19.9 - 21.0) | 3.0 |  | 5065 | 20.0 (19.4 - 20.6) | 3.1 |  | 5060 | 18.9 (18.4 - 19.4) | 3.0 |  | -5 | -1.1 (-1.9 to -0.3) | 0.94 (0.91 to 0.98) |
| Liver cancer (C22) | 39982 | 32.7 (32.4 - 33.0) |  | 7790 | 30.7 (30.0 - 31.3) | 4.5 |  | 7784 | 29.4 (28.8 - 30.1) | 4.5 |  | 7812 | 28.5 (27.8 - 29.1) | 4.5 |  | 28 | -0.9 (-1.9 to 0.0) | 0.97 (0.94 to 1.00) |
| Gallbladder cancer (C23) | 3786 | 3.3 (3.2 - 3.4) |  | 734 | 3.0 (2.8 - 3.2) | 0.4 |  | 805 | 3.2 (2.9 - 3.4) | 0.5 |  | 838 | 3.1 (2.9 - 3.3) | 0.5 |  | 33 | 0.0 (-0.3 to 0.3) | 0.99 (0.90 to 1.09) |
| Biliary tract cancer (C24) | 8061 | 7.1 (6.9 - 7.2) |  | 1770 | 7.3 (7.0 - 7.7) | 1.1 |  | 1763 | 7.0 (6.7 - 7.3) | 1.1 |  | 1946 | 7.3 (7.0 - 7.6) | 1.1 |  | 183 | 0.3 (-0.1 to 0.8) | 1.05 (0.98 to 1.12) |
| Pancreatic cancer (C25) | 15402 | 12.9 (12.7 - 13.1) |  | 3193 | 12.8 (12.4 - 13.3) | 1.9 |  | 3424 | 13.1 (12.7 - 13.5) | 2.0 |  | 3452 | 12.6 (12.2 - 13.0) | 2.0 |  | 28 | -0.5 (-1.1 to 0.1) | 0.96 (0.92 to 1.01) |
| Lung cancer (C33-C34) | 66159 | 57.5 (57.1 - 58.0) |  | 13188 | 54.5 (53.6 - 55.5) | 7.9 |  | 13698 | 53.8 (52.9 - 54.7) | 8.3 |  | 13824 | 51.7 (50.9 - 52.6) | 8.1 |  | 126 | -2.1 (-3.3 to -0.8) | 0.96 (0.94 to 0.98) |
| Prostate cancer (C61) | 9308 | 9.0 (8.8 - 9.2) |  | 1995 | 9.1 (8.7 - 9.5) | 1.3 |  | 2047 | 8.7 (8.3 - 9.1) | 1.3 |  | 2194 | 8.8 (8.4 - 9.1) | 1.4 |  | 147 | 0.1 (-0.5 to 0.6) | 1.01 (0.95 to 1.07) |
| Kidney cancer (C64) | 3421 | 2.9 (2.8 - 3.0) |  | 672 | 2.7 (2.5 - 2.9) | 0.4 |  | 666 | 2.6 (2.4 - 2.8) | 0.4 |  | 764 | 2.9 (2.7 - 3.1) | 0.4 |  | 98 | 0.3 (0.0 to 0.5) | 1.10 (0.99 to 1.22) |
| Bladder cancer (C67) | 5311 | 5.0 (4.9 - 5.1) |  | 1055 | 4.7 (4.4 - 5.0) | 0.7 |  | 1167 | 4.9 (4.6 - 5.2) | 0.8 |  | 1235 | 4.8 (4.6 - 5.1) | 0.8 |  | 68 | 0.0 (-0.4 to 0.4) | 0.99 (0.92 to 1.08) |
| Brain cancer (C70-72) | 3615 | 2.9 (2.8 - 3.0) |  | 727 | 2.9 (2.7 - 3.1) | 0.4 |  | 756 | 2.9 (2.7 - 3.1) | 0.4 |  | 784 | 2.9 (2.7 - 3.1) | 0.5 |  | 28 | 0.0 (-0.3 to 0.3) | 1.00 (0.91 to 1.11) |
| Non-Hodgkin's lymphoma (C82-C86) | 5462 | 4.6 (4.5 - 4.8) |  | 1177 | 4.8 (4.5 - 5.1) | 0.7 |  | 1169 | 4.6 (4.3 - 4.8) | 0.7 |  | 1192 | 4.5 (4.2 - 4.7) | 0.7 |  | 23 | -0.1 (-0.5 to 0.3) | 0.98 (0.90 to 1.06) |
| Multiple myeloma (C90) | 2540 | 2.2 (2.1 - 2.2) |  | 508 | 2.1 (1.9 - 2.2) | 0.3 |  | 527 | 2.1 (1.9 - 2.2) | 0.3 |  | 514 | 1.9 (1.7 - 2.1) | 0.3 |  | -13 | -0.2 (-0.4 to 0.1) | 0.92 (0.81 to 1.04) |
| Leukaemia (C91-C95) | 5294 | 4.4 (4.3 - 4.5) |  | 1081 | 4.4 (4.1 - 4.6) | 0.6 |  | 1143 | 4.4 (4.2 - 4.7) | 0.7 |  | 1048 | 3.9 (3.7 - 4.1) | 0.6 |  | -95 | -0.5 (-0.9 to -0.2) | 0.88 (0.81 to 0.96) |
| Other neoplasms(D00-D48) | 4090 | 3.6 (3.5 - 3.7) |  | **899** | **3.8 (3.5 - 4.0)** | **0.5** |  | 877 | 3.5 (3.3 - 3.8) | 0.5 |  | 869 | 3.3 (3.1 - 3.5) | 0.5 |  | -8 | -0.2 (-0.5 to 0.1) | 0.94 (0.86 to 1.04) |
| **Endocrine, nutritional, and metabolic diseases(E00-E90)** | **26513** | **23.5 (23.2 - 23.8)** |  | **5165** | **21.8 (21.2 - 22.4)** | **3.2** |  | **4817** | **19.3 (18.7 - 19.8)** | **3.0** |  | **5105** | **19.5 (19.0 - 20.0)** | **3.1** |  |  | **0.2 (-0.6 to 1.0)** | **1.01 (0.97 to 1.05)** |
| Diabetes mellitus (E10-E14) | 23199 | 20.6 (20.3 - 20.9) |  | 4423 | 18.7 (18.1 - 19.2) | 2.7 |  | 4121 | 16.5 (16.0 - 17.0) | 2.5 |  | 4320 | 16.5 (16.0 - 17.0) | 2.6 |  | 199 | 0.0 (-0.7 to 0.7) | 1.00 (0.96 to 1.04) |
| **Mental and behavioural disorders and nervous system diseases(F00-F99, G00-G99)** | **34455** | **32.6 (32.2 - 32.9)** |  | **7240** | **32.2 (31.4 - 32.9)** | **4.7** |  | **7135** | **29.9 (29.2 - 30.7)** | **4.6** |  | **7576** | **29.9 (29.2 - 30.6)** | **4.7** |  |  | **0.0 (-1.0 to 1.0)** | **1.00 (0.97 to 1.03)** |
| Dementia (F00-F03, G30) | 14466 | 15.3 (15.1 - 15.6) |  | 2980 | 14.6 (14.1 - 15.2) | 2.1 |  | 3114 | 14.1 (13.6 - 14.6) | 2.2 |  | 3329 | 13.9 (13.5 - 14.4) | 2.2 |  | 215 | -0.2 (-0.9 to 0.5) | 0.99 (0.94 to 1.04) |
| Alcoholism (F10) | 4018 | 3.1 (3.0 - 3.2) |  | 844 | 3.2 (2.9 - 3.4) | 0.5 |  | 825 | 3.0 (2.8 - 3.2) | 0.5 |  | 969 | 3.4 (3.2 - 3.7) | 0.5 |  | 144 | 0.4 (0.1 to 0.7) | 1.14 (1.04 to 1.25) |
| Parkinson's disease (G20) | 8153 | 7.7 (7.6 - 7.9) |  | 1775 | 7.9 (7.5 - 8.3) | 1.1 |  | 1549 | 6.5 (6.2 - 6.8) | 1.0 |  | 1643 | 6.5 (6.1 - 6.8) | 1.0 |  | 94 | 0.0 (-0.5 to 0.4) | 0.99 (0.93 to 1.07) |
| **Diseases of the circulatory system(I00-I99)** | **143320** | **131.1 (130.4 - 131.8)** |  | **29342** | **127.1 (125.6 - 128.6)** | **18.5** |  | **28672** | **117.7 (116.3 - 119.0)** | **18.1** |  | **29568** | **115.1 (113.7 - 116.4)** | **18.1** |  |  | **-2.6 (-4.5 to -0.7)** | **0.98 (0.96 to 0.99)** |
| Hypertensive diseases (I10-I15) | 8576 | 8.6 (8.4 - 8.8) |  | 1828 | 8.6 (8.2 - 9.0) | 1.2 |  | 1795 | 7.8 (7.4 - 8.2) | 1.2 |  | 1997 | 8.1 (7.8 - 8.5) | 1.3 |  | 202 | 0.3 (-0.2 to 0.8) | 1.04 (0.97 to 1.11) |
| Ischaemic heart diseases (I20-I25) | 39223 | 35.0 (34.7 - 35.4) |  | 7921 | 33.6 (32.8 - 34.3) | 4.9 |  | 7696 | 31.0 (30.3 - 31.7) | 4.8 |  | 7959 | 30.4 (29.7 - 31.1) | 4.8 |  | 263 | -0.5 (-1.5 to 0.4) | 0.98 (0.95 to 1.01) |
| Atrial fibrillation (I48) | 1950 | 2.0 (1.9 - 2.0) |  | 380 | 1.8 (1.6 - 2.0) | 0.3 |  | 422 | 1.9 (1.7 - 2.0) | 0.3 |  | 409 | 1.7 (1.5 - 1.8) | 0.3 |  | -13 | -0.2 (-0.4 to 0.1) | 0.90 (0.78 to 1.03) |
| Heart failure (I50) | 9442 | 9.5 (9.3 - 9.7) |  | 2240 | 10.5 (10.1 - 11.0) | 1.5 |  | 2268 | 9.9 (9.5 - 10.3) | 1.5 |  | 2420 | 9.9 (9.5 - 10.3) | 1.6 |  | 152 | 0.0 (-0.6 to 0.6) | 1.00 (0.95 to 1.06) |
| Cerebrovascular diseases (I60-I69) | 55551 | 50.7 (50.3 - 51.2) |  | 10927 | 47.0 (46.1 - 47.9) | 6.8 |  | 10626 | 43.5 (42.6 - 44.3) | 6.7 |  | 10630 | 41.2 (40.5 - 42.0) | 6.5 |  | 4 | -2.2 (-3.4 to -1.1) | 0.95 (0.92 to 0.98) |
| Haemorrhagic stroke (I60-I62) | 17792 | 15.2 (14.9 - 15.4) |  | 3516 | 14.4 (13.9 - 14.8) | 2.1 |  | 3561 | 14.0 (13.6 - 14.5) | 2.2 |  | 3534 | 13.4 (12.9 - 13.8) | 2.1 |  | -27 | -0.7 (-1.3 to 0.0) | 0.95 (0.91 to 1.00) |
| Ischaemic stroke (I63) | 17399 | 16.7 (16.5 - 17.0) |  | 3363 | 15.1 (14.6 - 15.6) | 2.2 |  | 3435 | 14.6 (14.1 - 15.1) | 2.2 |  | 3614 | 14.4 (13.9 - 14.9) | 2.3 |  | 179 | -0.2 (-0.9 to 0.5) | 0.99 (0.94 to 1.03) |
| Other stroke (I64-I69) | 20360 | 18.8 (18.6 - 19.1) |  | 4048 | 17.5 (17.0 - 18.1) | 2.6 |  | 3630 | 14.8 (14.4 - 15.3) | 2.3 |  | 3482 | 13.5 (13.0 - 14.0) | 2.1 |  | -148 | -1.3 (-2.0 to -0.7) | 0.91 (0.87 to 0.95) |
| Aortic aneurysm (I71) | 2889 | 2.6 (2.5 - 2.7) |  | 600 | 2.6 (2.3 - 2.8) | 0.4 |  | 627 | 2.5 (2.3 - 2.7) | 0.4 |  | 622 | 2.4 (2.2 - 2.6) | 0.4 |  | -5 | -0.2 (-0.4 to 0.1) | 0.94 (0.84 to 1.05) |
| **Diseases of the respiratory system(J00-J99)** | **91320** | **90.5 (89.9 - 91.1)** |  | **20777** | **96.6 (95.2 - 97.9)** | **14.1** |  | **20513** | **89.0 (87.8 - 90.3)** | **13.7** |  | **20671** | **83.5 (82.3 - 84.6)** | **13.1** |  |  | **-5.6 (-7.2 to -3.9)** | **0.94 (0.92 to 0.96)** |
| Pneumonia (J12-J18) | 50503 | 50.7 (50.3 - 51.2) |  | 12111 | 56.9 (55.9 - 58.0) | 8.3 |  | 12157 | 53.3 (52.4 - 54.3) | 8.2 |  | 12085 | 49.2 (48.3 - 50.0) | 7.7 |  | -72 | -4.2 (-5.5 to -2.8) | 0.92 (0.90 to 0.95) |
| Chronic lower respiratory diseases (J40-J47) | 21614 | 21.4 (21.1 - 21.7) |  | 4221 | 19.6 (19.0 - 20.2) | 2.9 |  | 4055 | 17.6 (17.0 - 18.1) | 2.7 |  | 3678 | 14.9 (14.4 - 15.3) | 2.3 |  | -377 | -2.7 (-3.5 to -2.0) | 0.85 (0.81 to 0.88) |
| Pneumonitis due to solids and liquids (J69) | 6393 | 6.4 (6.3 - 6.6) |  | 1515 | 7.1 (6.7 - 7.5) | 1.0 |  | 1525 | 6.7 (6.3 - 7.0) | 1.0 |  | 1906 | 7.8 (7.4 - 8.1) | 1.2 |  | 381 | 1.1 (0.6 to 1.6) | 1.16 (1.09 to 1.24) |
| Interstitial pulmonary diseases (J84) | 5332 | 4.8 (4.6 - 4.9) |  | 1143 | 4.9 (4.6 - 5.2) | 0.7 |  | 1180 | 4.7 (4.4 - 5.0) | 0.7 |  | 1185 | 4.5 (4.2 - 4.8) | 0.7 |  | 5 | -0.2 (-0.6 to 0.2) | 0.96 (0.88 to 1.04) |
| **Diseases of the digestive system(K00-K93)** | **37539** | **31.2 (30.9 - 31.5)** |  | **7650** | **30.6 (30.0 - 31.3)** | **4.5** |  | **7316** | **28.2 (27.5 - 28.8)** | **4.3** |  | **7922** | **29.4 (28.8 - 30.1)** | **4.6** |  |  | **1.2 (0.3 to 2.2)** | **1.04 (1.01 to 1.08)** |
| Liver diseases (K70-K77) | 25342 | 20.0 (19.7 - 20.2) |  | 5098 | 19.5 (19.0 - 20.1) | 2.8 |  | 4772 | 17.8 (17.3 - 18.3) | 2.7 |  | 5192 | 18.9 (18.4 - 19.4) | 3.0 |  | 420 | 1.1 (0.4 to 1.8) | 1.06 (1.02 to 1.11) |
| Alcoholic liver disease (K70) | 16277 | 12.6 (12.4 - 12.8) |  | 3276 | 12.3 (11.9 - 12.7) | 1.8 |  | 3140 | 11.6 (11.2 - 12.0) | 1.8 |  | 3354 | 12.0 (11.6 - 12.5) | 1.9 |  | 214 | 0.5 (-0.1 to 1.1) | 1.04 (0.99 to 1.09) |
| Liver cirrhosis (K74) | 7110 | 5.8 (5.6 - 5.9) |  | 1374 | 5.4 (5.1 - 5.7) | 0.8 |  | 1248 | 4.7 (4.4 - 5.0) | 0.7 |  | 1333 | 4.9 (4.6 - 5.2) | 0.8 |  | 85 | 0.2 (-0.2 to 0.6) | 1.04 (0.97 to 1.13) |
| **Diseases of the genitourinary system(N00-N99)** | **16862** | **15.9 (15.6 - 16.1)** |  | **3548** | **15.8 (15.3 - 16.3)** | **2.3** |  | **3712** | **15.6 (15.1 - 16.1)** | **2.4** |  | **4113** | **16.3 (15.8 - 16.8)** | **2.6** |  |  | **0.7 (0.0 to 1.4)** | **1.04 (1.00 to 1.09)** |
| Renal failure (N17-19) | 13734 | 12.8 (12.6 - 13.0) |  | 2850 | 12.6 (12.1 - 13.1) | 1.8 |  | 2990 | 12.5 (12.0 - 12.9) | 1.9 |  | 3323 | 13.0 (12.6 - 13.5) | 2.0 |  | 333 | 0.6 (-0.1 to 1.2) | 1.05 (1.00 to 1.10) |
| **Symptoms, signs, and abnormal clinical and laboratory findings, NEC(R00-R99)** | **57283** | **54.8 (54.3 - 55.2)** |  | **12414** | **55.8 (54.8 - 56.8)** | **8.1** |  | **12542** | **52.9 (51.9 - 53.8)** | **8.2** |  | **14135** | **56.4 (55.5 - 57.4)** | **8.9** |  |  | **3.6 (2.2 to 4.9)** | **1.07 (1.04 to 1.09)** |
| Senility (R54) | 21850 | 24.2 (23.9 - 24.5) |  | 4428 | 22.7 (22.0 - 23.4) | 3.3 |  | 4210 | 19.8 (19.2 - 20.4) | 3.0 |  | 5150 | 22.2 (21.6 - 22.8) | 3.5 |  | 940 | 2.4 (1.6 to 3.3) | 1.12 (1.08 to 1.17) |
| Other ill-defined and unspecified causes of mortality (R99) | 23035 | 19.3 (19.0 - 19.5) |  | 5122 | 20.6 (20.0 - 21.2) | 3.0 |  | 5391 | 21.0 (20.5 - 21.6) | 3.2 |  | 5602 | 21.1 (20.5 - 21.6) | 3.3 |  | 211 | 0.0 (-0.8 to 0.8) | 1.00 (0.96 to 1.04) |
| **External causes of morbidity and mortality(V01-Y98)** | **95946** | **78.5 (78.0 - 79.0)** |  | **19493** | **77.5 (76.4 - 78.6)** | **11.3** |  | **18896** | **73.1 (72.0 - 74.1)** | **11.3** |  | **18032** | **68.2 (67.2 - 69.2)** | **10.7** |  |  | **-4.9 (-6.4 to -3.5)** | **0.93 (0.91 to 0.95)** |
| Transport Accidents (V01-V99) | 18096 | 14.6 (14.4 - 14.9) |  | 3391 | 13.3 (12.9 - 13.8) | 1.9 |  | 3162 | 12.1 (11.7 - 12.6) | 1.9 |  | 2955 | 11.0 (10.6 - 11.4) | 1.7 |  | -207 | -1.1 (-1.7 to -0.5) | 0.91 (0.86 to 0.95) |
| Falls (W00-W19) | 9370 | 7.8 (7.6 - 7.9) |  | 1915 | 7.7 (7.3 - 8.0) | 1.1 |  | 1928 | 7.5 (7.2 - 7.8) | 1.2 |  | 1926 | 7.2 (6.8 - 7.5) | 1.1 |  | -2 | -0.3 (-0.8 to 0.1) | 0.96 (0.90 to 1.02) |
| Intentional self-harm (X60-X84) | 47316 | 37.8 (37.4 - 38.1) |  | 9862 | 38.5 (37.8 - 39.3) | 5.6 |  | 9730 | 37.3 (36.6 - 38.1) | 5.8 |  | 9093 | 34.5 (33.8 - 35.3) | 5.4 |  | -637 | -2.8 (-3.8 to -1.8) | 0.93 (0.90 to 0.95) |
| **Sub-specific causes groups** |  |  |  |  |  |  |  |  |  |  |  |  |  |  |  |  |  |  |
| Alcohol-specific disorders and poisonings | 20900 | 16.1 (15.9 - 16.3) |  | 4262 | 16.0 (15.5 - 16.5) | 2.3 |  | 4084 | 15.0 (14.6 - 15.5) | 2.3 |  | 4459 | 16.0 (15.5 - 16.5) | 2.5 |  | 375 | 1.0 (0.3 to 1.6) | 1.06 (1.02 to 1.11) |
| Smoking-related causes (+35 years) | 424444 | 381.5 (380.4-382.7) |  | 87002 | 371.9 (369.4 - 374.4) | 54.1 |  | 86831 | 351.1 (348.8-353.5) | 54.1 |  | 87599 | 335.5 (333.2 - 337.7) | 52.7 |  | 768 | -26.5 (-32.0 to -21.0) | 0.96 (0.95 to 0.96) |
| Avoidable causes (0-74 years) | 304624 | 234.6 (233.8-235.4) |  | 59644 | 224.4 (222.6 - 226.2) | 32.7 |  | 58665 | 214.2 (212.5-215.9) | 33.0 |  | 58339 | 206.2 (204.5 - 207.9) | 32.4 |  | -326 | -8.4 (-11.0 to -5.9) | 0.96 (0.95 to 0.97) |
| Amenable causes | 107761 | 83.4 (82.9-83.9) |  | 21340 | 80.5 (79.4 - 81.6) | 11.7 |  | 20793 | 75.8 (74.8-76.9) | 11.7 |  | 20999 | 73.6 (72.6 - 74.6) | 11.6 |  | 206 | -2.3 (-3.9 to -0.8) | 0.97 (0.95 to 0.99) |
| Preventable causes | 260817 | 200.6 (199.8-201.3) |  | 50649 | 190.3 (188.6 - 191.9) | 27.7 |  | 49725 | 181.4 (179.8-183.0) | 28.0 |  | 49148 | 174.0 (172.4 - 175.5) | 27.3 |  | -577 | -7.9 (-10.2 to -5.5) | 0.96 (0.95 to 0.97) |
