# Supplementary material for: Cause-specific mortality in Korea during the first year of the COVID-19 pandemic
Source: Epidemiol Health. 2022 Nov 23;44:e2022110. doi: 10.4178/epih.e2022110 (PMC10106553; doi:10.4178/epih.e2022110)
Supplement: Supplementary file 5 [file epih-44-e2022110-Supplementary-5.docx]

Supplementary Material 5. Age-standardized death rates by specific causes of death in 2015-2019 (combined), 2018, 2019, and 2020 among Korean women

| **Causes** | 2015-2019 combined | |  | 2018 | | |  | 2019 | | |  | 2020 | | |  | Between 2019 and 2020 | | |
| --- | --- | --- | --- | --- | --- | --- | --- | --- | --- | --- | --- | --- | --- | --- | --- | --- | --- | --- |
|  | No of deaths | Age-standardized mortality rates  (per 100,000) |  | No of deaths | Age-standardized mortality rates  (per 100,000) | % |  | No of deaths | Age-standardized mortality rates  (per 100,000) | % |  | No of deaths | Age-standardized mortality rates  (per 100,000) | % |  | Number difference | Rate difference | Rate ratio |
| **Certain infectious and parasitic diseases(A00-B99, U07.1, U07.2, U08-U10)** | **20789** | **11.3 (11.1 - 11.4)** |  | **4537** | **11.6 (11.3 - 12.0)** | **3.2** |  | **4589** | **11.1 (10.7 - 11.4)** | **3.2** |  | **5529** | **12.6 (12.3 - 13.0)** | **3.7** |  |  | **1.6 (1.1 to 2.1)** | **1.14 (1.10 to 1.19)** |
| Tuberculosis (A15-A19) | 3678 | 2.0 (1.9 - 2.0) |  | 680 | 1.7 (1.6 - 1.8) | 0.5 |  | 633 | 1.5 (1.4 - 1.6) | 0.4 |  | 514 | 1.2 (1.1 - 1.3) | 0.3 |  | -119 | -0.3 (-0.5 to -0.2) | 0.78 (0.69 to 0.88) |
| Sepsis (A40-A41) | 11795 | 6.3 (6.2 - 6.4) |  | 2719 | 6.8 (6.6 - 7.1) | 1.9 |  | 2890 | 6.8 (6.5 - 7.1) | 2.0 |  | 3533 | 7.9 (7.6 - 8.2) | 2.3 |  | 643 | 1.1 (0.7 to 1.5) | 1.16 (1.10 to 1.22) |
| Covid19 (U07.1, U07.2, U08-U10) | 0 | 0 |  | 0 | 0 | 0.0 |  | 0 | 0 | 0.0 |  | 467 | 1.1 (1.0 - 1.2) | 0.3 |  | 467 | - | - |
| **Malignant neoplasms(C00-C97)** | **150337** | **94.1 (93.6 - 94.5)** |  | **30255** | **90.6 (89.5 - 91.6)** | **24.7** |  | **30922** | **89.3 (88.3 - 90.3)** | **26.1** |  | **31387** | **87.5 (86.5 - 88.5)** | **25.9** |  |  | **-1.8 (-3.2 to -0.3)** | **0.98 (0.96 to 1.00)** |
| Oesophageal cancer (C15) | 658 | 0.4 (0.4 - 0.4) |  | 121 | 0.4 (0.3 - 0.4) | 0.1 |  | 129 | 0.4 (0.3 - 0.4) | 0.1 |  | 160 | 0.5 (0.4 - 0.5) | 0.1 |  | 31 | 0.1 (0.0 to 0.2) | 1.22 (0.96 to 1.54) |
| Stomach cancer (C16) | 14164 | 8.7 (8.5 - 8.8) |  | 2663 | 7.8 (7.4 - 8.1) | 2.1 |  | 2668 | 7.5 (7.2 - 7.8) | 2.2 |  | 2703 | 7.4 (7.1 - 7.7) | 2.2 |  | 35 | -0.1 (-0.5 to 0.3) | 0.99 (0.94 to 1.05) |
| Colorectal cancer (C18-C21) | 18916 | 11.3 (11.1 - 11.4) |  | 3863 | 11.0 (10.6 - 11.3) | 3.0 |  | 3901 | 10.6 (10.3 - 11.0) | 3.1 |  | 3884 | 10.1 (9.8 - 10.4) | 3.0 |  | -17 | -0.5 (-1.0 to 0.0) | 0.95 (0.91 to 1.00) |
| Liver cancer (C22) | 14248 | 8.9 (8.7 - 9.0) |  | 2821 | 8.3 (8.0 - 8.6) | 2.3 |  | 2802 | 8.0 (7.7 - 8.3) | 2.3 |  | 2753 | 7.5 (7.2 - 7.8) | 2.2 |  | -49 | -0.5 (-0.9 to -0.1) | 0.93 (0.89 to 0.99) |
| Gallbladder cancer (C23) | 5102 | 3.1 (3.0 - 3.2) |  | 1004 | 2.9 (2.7 - 3.1) | 0.8 |  | 1101 | 3.0 (2.8 - 3.2) | 0.9 |  | 1052 | 2.8 (2.6 - 2.9) | 0.8 |  | -49 | -0.2 (-0.5 to 0.0) | 0.92 (0.85 to 1.01) |
| Biliary tract cancer (C24) | 6246 | 3.6 (3.5 - 3.7) |  | 1363 | 3.8 (3.6 - 4.0) | 1.0 |  | 1319 | 3.5 (3.3 - 3.7) | 1.0 |  | 1356 | 3.4 (3.2 - 3.6) | 1.0 |  | 37 | -0.1 (-0.3 to 0.2) | 0.98 (0.91 to 1.06) |
| Pancreatic cancer (C25) | 13865 | 8.5 (8.4 - 8.7) |  | 2843 | 8.4 (8.1 - 8.7) | 2.3 |  | 2972 | 8.4 (8.1 - 8.7) | 2.5 |  | 3323 | 9.0 (8.7 - 9.3) | 2.7 |  | 351 | 0.6 (0.2 to 1.0) | 1.07 (1.02 to 1.13) |
| Lung cancer (C33-C34) | 23609 | 14.3 (14.1 - 14.5) |  | 4664 | 13.5 (13.1 - 13.9) | 3.7 |  | 4876 | 13.5 (13.1 - 13.9) | 3.9 |  | 4849 | 12.9 (12.5 - 13.2) | 3.8 |  | -27 | -0.6 (-1.2 to -0.1) | 0.95 (0.92 to 0.99) |
| Breast cancer (C50), Female | 12373 | 8.8 (8.7 - 9.0) |  | 2460 | 8.5 (8.2 - 8.9) | 2.3 |  | 2622 | 8.9 (8.6 - 9.3) | 2.6 |  | 2725 | 9.1 (8.7 - 9.4) | 2.7 |  | 103 | 0.1 (-0.4 to 0.6) | 1.02 (0.96 to 1.07) |
| Cervical cancer (C53) | 4475 | 3.0 (2.9 - 3.1) |  | 845 | 2.8 (2.6 - 3.0) | 0.8 |  | 898 | 2.9 (2.7 - 3.1) | 0.8 |  | 810 | 2.5 (2.4 - 2.7) | 0.8 |  | -88 | -0.3 (-0.6 to -0.1) | 0.89 (0.80 to 0.98) |
| Uterus cancer (C54-C55) | 2087 | 1.4 (1.4 - 1.5) |  | 423 | 1.4 (1.2 - 1.5) | 0.4 |  | 443 | 1.4 (1.3 - 1.6) | 0.4 |  | 474 | 1.5 (1.3 - 1.6) | 0.4 |  | 31 | 0.0 (-0.2 to 0.2) | 1.03 (0.90 to 1.17) |
| Ovarian cancer (C56) | 5885 | 4.0 (3.9 - 4.1) |  | 1243 | 4.1 (3.9 - 4.3) | 1.1 |  | 1234 | 4.0 (3.8 - 4.2) | 1.2 |  | 1369 | 4.3 (4.1 - 4.5) | 1.3 |  | 135 | 0.3 (0.0 to 0.6) | 1.08 (1.00 to 1.17) |
| Kidney cancer (C64) | 1535 | 0.9 (0.9 - 1.0) |  | 317 | 0.9 (0.8 - 1.0) | 0.3 |  | 317 | 0.9 (0.8 - 1.0) | 0.3 |  | 312 | 0.8 (0.7 - 0.9) | 0.2 |  | -5 | 0.0 (-0.2 to 0.1) | 0.96 (0.81 to 1.12) |
| Bladder cancer (C67) | 1765 | 1.0 (0.9 - 1.0) |  | 345 | 0.9 (0.8 - 1.0) | 0.2 |  | 383 | 1.0 (0.9 - 1.0) | 0.3 |  | 358 | 0.9 (0.8 - 1.0) | 0.3 |  | -25 | -0.1 (-0.2 to 0.0) | 0.91 (0.78 to 1.05) |
| Brain cancer (C70-72) | 3068 | 2.1 (2.0 - 2.2) |  | 628 | 2.1 (1.9 - 2.3) | 0.6 |  | 660 | 2.1 (2.0 - 2.3) | 0.6 |  | 653 | 2.0 (1.9 - 2.2) | 0.6 |  | -7 | -0.1 (-0.3 to 0.1) | 0.96 (0.85 to 1.07) |
| Non-Hodgkin's lymphoma (C82-C86) | 3989 | 2.5 (2.4 - 2.6) |  | 856 | 2.6 (2.4 - 2.7) | 0.7 |  | 846 | 2.5 (2.3 - 2.6) | 0.7 |  | 877 | 2.4 (2.3 - 2.6) | 0.7 |  | 31 | 0.0 (-0.3 to 0.2) | 0.99 (0.90 to 1.09) |
| Multiple myeloma (C90) | 2237 | 1.4 (1.3 - 1.5) |  | 445 | 1.3 (1.2 - 1.4) | 0.4 |  | 434 | 1.2 (1.1 - 1.4) | 0.4 |  | 462 | 1.3 (1.1 - 1.4) | 0.4 |  | 28 | 0.0 (-0.1 to 0.2) | 1.02 (0.89 to 1.16) |
| Leukaemia (C91-C95) | 3861 | 2.6 (2.5 - 2.7) |  | 767 | 2.5 (2.3 - 2.6) | 0.7 |  | 768 | 2.4 (2.2 - 2.5) | 0.7 |  | 777 | 2.4 (2.2 - 2.5) | 0.7 |  | 9 | 0.0 (-0.2 to 0.2) | 1.00 (0.90 to 1.11) |
| Other neoplasms(D00-D48) | 3563 | 2.1 (2.0 - 2.2) |  | **695** | **2.0 (1.8 - 2.1)** | **0.5** |  | 764 | 2.1 (1.9 - 2.2) | 0.6 |  | 703 | 1.8 (1.7 - 2.0) | 0.5 |  | -61 | -0.3 (-0.5 to 0.0) | 0.88 (0.79 to 0.98) |
| **Endocrine, nutritional, and metabolic diseases(E00-E90)** | **26567** | **14.8 (14.6 - 15.0)** |  | **5159** | **13.6 (13.2 - 14.0)** | **3.7** |  | **4685** | **11.7 (11.3 - 12.0)** | **3.4** |  | **4947** | **11.7 (11.4 - 12.0)** | **3.5** |  |  | **0.0 (-0.5 to 0.5)** | **1.00 (0.96 to 1.04)** |
| Diabetes mellitus (E10-E14) | 23241 | 12.9 (12.8 - 13.1) |  | 4366 | 11.5 (11.2 - 11.8) | 3.1 |  | 3981 | 9.9 (9.6 - 10.2) | 2.9 |  | 4136 | 9.8 (9.5 - 10.1) | 2.9 |  | 155 | -0.1 (-0.6 to 0.3) | 0.99 (0.94 to 1.03) |
| **Mental and behavioural disorders and nervous system diseases(F00-F99, G00-G99)** | **49352** | **25.6 (25.4 - 25.8)** |  | **10199** | **25.1 (24.6 - 25.6)** | **6.8** |  | **10440** | **24.0 (23.5 - 24.5)** | **7.0** |  | **10562** | **22.9 (22.4 - 23.3)** | **6.8** |  |  | **-1.1 (-1.8 to -0.5)** | **0.95 (0.93 to 0.98)** |
| Dementia (F00-F03, G30) | 33545 | 16.3 (16.1 - 16.5) |  | 6759 | 15.4 (15.0 - 15.8) | 4.2 |  | 7243 | 15.4 (15.0 - 15.7) | 4.5 |  | 7312 | 14.4 (14.1 - 14.8) | 4.3 |  | 69 | -1.0 (-1.5 to -0.5) | 0.94 (0.91 to 0.97) |
| Alcoholism (F10) | 451 | 0.3 (0.3 - 0.4) |  | 96 | 0.4 (0.3 - 0.4) | 0.1 |  | 91 | 0.3 (0.3 - 0.4) | 0.1 |  | 120 | 0.4 (0.4 - 0.5) | 0.1 |  | 29 | 0.1 (0.0 to 0.2) | 1.30 (0.98 to 1.71) |
| Parkinson's disease (G20) | 9976 | 5.5 (5.4 - 5.6) |  | 2173 | 5.6 (5.4 - 5.9) | 1.5 |  | 1927 | 4.7 (4.5 - 4.9) | 1.4 |  | 1928 | 4.5 (4.3 - 4.7) | 1.3 |  | 1 | -0.2 (-0.5 to 0.1) | 0.95 (0.89 to 1.02) |
| **Diseases of the circulatory system(I00-I99)** | **161076** | **86.1 (85.7 - 86.6)** |  | **33605** | **84.6 (83.7 - 85.5)** | **23.1** |  | **31580** | **75.3 (74.4 - 76.1)** | **22.0** |  | **32628** | **73.3 (72.5 - 74.1)** | **21.7** |  |  | **-2.0 (-3.2 to -0.8)** | **0.97 (0.96 to 0.99)** |
| Hypertensive diseases (I10-I15) | 19362 | 9.6 (9.5 - 9.7) |  | 4237 | 9.9 (9.6 - 10.2) | 2.7 |  | 3836 | 8.4 (8.1 - 8.6) | 2.4 |  | 4103 | 8.4 (8.1 - 8.7) | 2.5 |  | 267 | 0.0 (-0.4 to 0.4) | 1.00 (0.96 to 1.05) |
| Ischaemic heart diseases (I20-I25) | 32611 | 17.4 (17.2 - 17.6) |  | 6579 | 16.5 (16.1 - 16.9) | 4.5 |  | 6003 | 14.3 (14.0 - 14.7) | 4.2 |  | 6097 | 13.8 (13.4 - 14.2) | 4.1 |  | 94 | -0.5 (-1.0 to 0.0) | 0.96 (0.93 to 1.00) |
| Atrial fibrillation (I48) | 3402 | 1.7 (1.7 - 1.8) |  | 654 | 1.6 (1.4 - 1.7) | 0.4 |  | 731 | 1.6 (1.5 - 1.8) | 0.5 |  | 735 | 1.5 (1.4 - 1.6) | 0.5 |  | 4 | -0.1 (-0.3 to 0.1) | 0.94 (0.85 to 1.04) |
| Heart failure (I50) | 19639 | 9.9 (9.7 - 10.0) |  | 4515 | 10.7 (10.4 - 11.0) | 2.9 |  | 4490 | 9.9 (9.6 - 10.2) | 2.9 |  | 4836 | 10.0 (9.7 - 10.3) | 3.0 |  | 346 | 0.1 (-0.3 to 0.5) | 1.01 (0.97 to 1.05) |
| Cerebrovascular diseases (I60-I69) | 59590 | 32.8 (32.5 - 33.1) |  | 12013 | 31.2 (30.6 - 31.8) | 8.5 |  | 10960 | 27.2 (26.7 - 27.7) | 7.9 |  | 11230 | 26.3 (25.8 - 26.8) | 7.8 |  | 270 | -0.9 (-1.6 to -0.2) | 0.97 (0.94 to 0.99) |
| Haemorrhagic stroke (I60-I62) | 17381 | 10.5 (10.4 - 10.7) |  | 3351 | 9.7 (9.4 - 10.0) | 2.6 |  | 3381 | 9.5 (9.1 - 9.8) | 2.8 |  | 3558 | 9.5 (9.2 - 9.8) | 2.8 |  | 177 | 0.0 (-0.4 to 0.5) | 1.00 (0.95 to 1.05) |
| Ischaemic stroke (I63) | 19568 | 10.2 (10.1 - 10.4) |  | 3959 | 9.7 (9.4 - 10.0) | 2.6 |  | 3668 | 8.4 (8.2 - 8.7) | 2.5 |  | 3800 | 8.2 (7.9 - 8.4) | 2.4 |  | 132 | -0.3 (-0.6 to 0.1) | 0.97 (0.93 to 1.02) |
| Other stroke (I64-I69) | 22641 | 12.1 (11.9 - 12.2) |  | 4703 | 11.8 (11.5 - 12.1) | 3.2 |  | 3911 | 9.3 (9.0 - 9.6) | 2.7 |  | 3872 | 8.6 (8.3 - 8.9) | 2.6 |  | -39 | -0.7 (-1.1 to -0.3) | 0.93 (0.89 to 0.97) |
| Aortic aneurysm (I71) | 2387 | 1.4 (1.3 - 1.4) |  | 510 | 1.4 (1.3 - 1.5) | 0.4 |  | 520 | 1.3 (1.2 - 1.5) | 0.4 |  | 484 | 1.2 (1.1 - 1.3) | 0.4 |  | -36 | -0.2 (-0.3 to 0.0) | 0.88 (0.77 to 1.00) |
| **Diseases of the respiratory system(J00-J99)** | **72952** | **37.5 (37.2 - 37.7)** |  | **16986** | **41.1 (40.4 - 41.7)** | **11.2** |  | **16142** | **36.7 (36.1 - 37.3)** | **10.7** |  | **15697** | **33.7 (33.2 - 34.3)** | **10.0** |  |  | **-3.0 (-3.8 to -2.2)** | **0.92 (0.90 to 0.94)** |
| Pneumonia (J12-J18) | 46517 | 23.6 (23.4 - 23.8) |  | 11169 | 26.7 (26.1 - 27.2) | 7.3 |  | 11011 | 24.7 (24.3 - 25.2) | 7.2 |  | 10172 | 21.6 (21.2 - 22.0) | 6.4 |  | -839 | -3.1 (-3.8 to -2.5) | 0.87 (0.85 to 0.90) |
| Chronic lower respiratory diseases (J40-J47) | 12450 | 6.4 (6.3 - 6.5) |  | 2387 | 5.8 (5.5 - 6.0) | 1.6 |  | 2121 | 4.8 (4.6 - 5.0) | 1.4 |  | 1990 | 4.2 (4.0 - 4.4) | 1.2 |  | -131 | -0.6 (-0.9 to -0.3) | 0.88 (0.82 to 0.93) |
| Pneumonitis due to solids and liquids (J69) | 4914 | 2.5 (2.5 - 2.6) |  | 1133 | 2.8 (2.6 - 2.9) | 0.8 |  | 1106 | 2.5 (2.4 - 2.7) | 0.7 |  | 1476 | 3.2 (3.0 - 3.3) | 0.9 |  | 370 | 0.7 (0.4 to 0.9) | 1.26 (1.16 to 1.37) |
| Interstitial pulmonary diseases (J84) | 3094 | 1.8 (1.7 - 1.9) |  | 681 | 1.9 (1.8 - 2.0) | 0.5 |  | 633 | 1.7 (1.5 - 1.8) | 0.5 |  | 621 | 1.5 (1.4 - 1.7) | 0.5 |  | -12 | -0.1 (-0.3 to 0.1) | 0.93 (0.83 to 1.04) |
| **Diseases of the digestive system(K00-K93)** | **22615** | **13.1 (12.9 - 13.3)** |  | **4751** | **13.1 (12.7 - 13.5)** | **3.6** |  | **4647** | **12.3 (11.9 - 12.7)** | **3.6** |  | **4948** | **12.5 (12.1 - 12.9)** | **3.7** |  |  | **0.2 (-0.3 to 0.7)** | **1.01 (0.97 to 1.06)** |
| Liver diseases (K70-K77) | 8454 | 5.5 (5.4 - 5.7) |  | 1760 | 5.6 (5.3 - 5.9) | 1.5 |  | 1724 | 5.3 (5.0 - 5.6) | 1.5 |  | 1787 | 5.4 (5.1 - 5.7) | 1.6 |  | 63 | 0.1 (-0.3 to 0.5) | 1.02 (0.95 to 1.09) |
| Alcoholic liver disease (K70) | 2556 | 1.9 (1.9 - 2.0) |  | 542 | 2.0 (1.9 - 2.2) | 0.6 |  | 518 | 1.9 (1.8 - 2.1) | 0.6 |  | 587 | 2.2 (2.0 - 2.4) | 0.6 |  | 69 | 0.2 (0.0 to 0.5) | 1.12 (0.99 to 1.26) |
| Liver cirrhosis (K74) | 4425 | 2.7 (2.7 - 2.8) |  | 914 | 2.7 (2.5 - 2.9) | 0.7 |  | 885 | 2.5 (2.3 - 2.7) | 0.7 |  | 869 | 2.3 (2.2 - 2.5) | 0.7 |  | -16 | -0.2 (-0.4 to 0.1) | 0.94 (0.85 to 1.03) |
| **Diseases of the genitourinary system(N00-N99)** | **21159** | **11.6 (11.4 - 11.7)** |  | **4612** | **11.8 (11.5 - 12.2)** | **3.2** |  | **4853** | **11.7 (11.4 - 12.1)** | **3.4** |  | **5230** | **11.9 (11.6 - 12.3)** | **3.5** |  |  | **0.2 (-0.3 to 0.7)** | **1.02 (0.98 to 1.06)** |
| Renal failure (N17-19) | 13468 | 7.5 (7.4 - 7.6) |  | 2807 | 7.4 (7.1 - 7.6) | 2.0 |  | 3046 | 7.5 (7.3 - 7.8) | 2.2 |  | 3266 | 7.6 (7.4 - 7.9) | 2.3 |  | 220 | 0.1 (-0.3 to 0.5) | 1.01 (0.96 to 1.07) |
| **Symptoms, signs, and abnormal clinical and laboratory findings, NEC(R00-R99)** | **74273** | **37.4 (37.1 - 37.6)** |  | **16052** | **38.3 (37.7 - 38.9)** | **10.5** |  | **15634** | **35.2 (34.6 - 35.8)** | **10.3** |  | **17666** | **37.0 (36.4 - 37.5)** | **11.0** |  |  | **1.8 (1.0 to 2.6)** | **1.05 (1.03 to 1.07)** |
| Senility (R54) | 48207 | 22.9 (22.7 - 23.1) |  | 9941 | 22.1 (21.6 - 22.5) | 6.0 |  | 9312 | 19.3 (18.9 - 19.7) | 5.6 |  | 10673 | 20.4 (20.0 - 20.8) | 6.1 |  | 1361 | 1.1 (0.6 to 1.7) | 1.06 (1.03 to 1.09) |
| Other ill-defined and unspecified causes of mortality (R99) | 12619 | 7.5 (7.3 - 7.6) |  | 2945 | 8.4 (8.1 - 8.7) | 2.3 |  | 3158 | 8.5 (8.2 - 8.8) | 2.5 |  | 3541 | 8.8 (8.5 - 9.1) | 2.6 |  | 383 | 0.3 (-0.1 to 0.8) | 1.04 (0.99 to 1.09) |
| **External causes of morbidity and mortality(V01-Y98)** | **43532** | **28.9 (28.7 - 29.2)** |  | **8547** | **27.9 (27.3 - 28.5)** | **7.6** |  | **8386** | **27.1 (26.5 - 27.7)** | **7.9** |  | **8410** | **26.6 (26.0 - 27.2)** | **7.9** |  |  | **-0.6 (-1.4 to 0.3)** | **0.98 (0.95 to 1.01)** |
| Transport Accidents (V01-V99) | 6513 | 4.4 (4.3 - 4.5) |  | 1280 | 4.2 (3.9 - 4.4) | 1.1 |  | 1059 | 3.3 (3.1 - 3.5) | 1.0 |  | 992 | 3.0 (2.8 - 3.2) | 0.9 |  | -67 | -0.3 (-0.6 to 0.0) | 0.90 (0.83 to 0.99) |
| Falls (W00-W19) | 3559 | 2.1 (2.0 - 2.2) |  | 754 | 2.2 (2.0 - 2.3) | 0.6 |  | 737 | 2.0 (1.8 - 2.1) | 0.6 |  | 737 | 1.9 (1.7 - 2.0) | 0.6 |  | 0 | -0.1 (-0.3 to 0.1) | 0.93 (0.84 to 1.04) |
| Intentional self-harm (X60-X84) | 19221 | 14.3 (14.1 - 14.5) |  | 3808 | 14.1 (13.7 - 14.6) | 3.9 |  | 4069 | 15.1 (14.6 - 15.5) | 4.4 |  | 4102 | 15.2 (14.7 - 15.6) | 4.5 |  | 33 | 0.1 (-0.6 to 0.8) | 1.01 (0.96 to 1.05) |
| **Sub-specific causes groups** |  |  |  |  |  |  |  |  |  |  |  |  |  |  |  |  |  |  |
| Alcohol-specific disorders and poisonings | 3162 | 2.4 (2.3 - 2.5) |  | 681 | 2.6 (2.4 - 2.8) | 0.7 |  | 641 | 2.4 (2.2 - 2.6) | 0.7 |  | 732 | 2.7 (2.5 - 2.9) | 0.8 |  | 91 | 0.3 (0.0 to 0.6) | 1.13 (1.02 to 1.26) |
| Smoking-related causes (+35 years) | 316886 | 176.3 (175.7-176.9) |  | 66045 | 173.2 (171.8 - 174.5) | 47.3 |  | 63876 | 159.6 (158.3-160.8) | 46.6 |  | 64129 | 152.4 (151.2 - 153.7) | 45.2 |  | 253 | -12.1 (-15.1 to -9.1) | 0.96 (0.94 to 0.97) |
| Avoidable causes (0-74 years) | 120219 | 89.2 (88.7-89.7) |  | 23107 | 84.8 (83.7 - 85.9) | 23.2 |  | 22888 | 82.5 (81.4-83.5) | 24.1 |  | 23034 | 81.1 (80.0 - 82.1) | 24.1 |  | 146 | -1.5 (-3.1 to 0.1) | 0.98 (0.97 to 1.00) |
| Amenable causes | 62789 | 46.1 (45.7-46.5) |  | 12039 | 43.6 (42.8 - 44.4) | 11.9 |  | 11743 | 41.6 (40.9-42.4) | 12.1 |  | 11844 | 40.7 (40.0 - 41.5) | 12.1 |  | 101 | -0.9 (-2.1 to 0.2) | 0.98 (0.95 to 1.00) |
| Preventable causes | 85937 | 63.8 (63.4-64.2) |  | 16324 | 60.0 (59.1 - 60.9) | 16.4 |  | 16066 | 58.0 (57.1-58.9) | 16.9 |  | 16106 | 56.9 (56.0 - 57.8) | 16.9 |  | 40 | -1.2 (-2.5 to 0.2) | 0.98 (0.96 to 1.00) |
