# Supplementary material for: Cause-specific mortality in Korea during the first year of the COVID-19 pandemic
Source: Epidemiol Health. 2022 Nov 23;44:e2022110. doi: 10.4178/epih.e2022110 (PMC10106553; doi:10.4178/epih.e2022110)
Supplement: Supplementary file 6 [file epih-44-e2022110-Supplementary-6.docx]

Supplementary Material 6. Age-specific mortality rates and differences between 2019 and 2020 by specific causes among Korean

|  | 2019 | |  | 2020 | |  |  | Between 2019 and 2020 | |
| --- | --- | --- | --- | --- | --- | --- | --- | --- | --- |
|  | No of deaths | Age-specific mortality rates  (per 100,000) |  | No of deaths | Age-specific mortality rates  (per 100,000) |  | Number difference | Rate difference | Rate ratio |
| Total |  |  |  |  |  |  |  |  |  |
| 0-4 | 1055 | 55 (52 - 59) |  | 859 | 49 (46 - 52) |  | -196 | -6.5 (-11.1 to -1.8) | 0.88 (0.81 to 0.97) |
| 5-9 | 178 | 8 (7 - 9) |  | 142 | 6 (5 - 7) |  | -36 | -1.5 (-3.0 to 0.0) | 0.80 (0.64 to 1.00) |
| 10-14 | 184 | 8 (7 - 9) |  | 203 | 9 (8 - 10) |  | 19 | 0.8 (-0.9 to 2.5) | 1.10 (0.90 to 1.34) |
| 15-19 | 610 | 22 (21 - 24) |  | 563 | 22 (20 - 24) |  | -47 | -0.3 (-2.9 to 2.2) | 0.99 (0.88 to 1.11) |
| 20-24 | 1126 | 34 (32 - 36) |  | 1118 | 34 (32 - 36) |  | -8 | 0.6 (-2.2 to 3.4) | 1.02 (0.94 to 1.11) |
| 25-29 | 1435 | 42 (40 - 44) |  | 1588 | 45 (43 - 48) |  | 153 | 3.5 (0.4 to 6.6) | 1.08 (1.01 to 1.16) |
| 30-34 | 1788 | 57 (54 - 59) |  | 1752 | 56 (53 - 59) |  | -36 | -0.8 (-4.5 to 2.9) | 0.99 (0.92 to 1.05) |
| 35-39 | 3117 | 79 (76 - 82) |  | 3007 | 79 (77 - 82) |  | -110 | 0.7 (-3.3 to 4.7) | 1.01 (0.96 to 1.06) |
| 40-44 | 4219 | 109 (106 - 113) |  | 4155 | 108 (104 - 111) |  | -64 | -1.8 (-6.5 to 2.8) | 0.98 (0.94 to 1.03) |
| 45-49 | 7701 | 172 (168 - 176) |  | 7419 | 169 (166 - 173) |  | -282 | -2.6 (-8.0 to 2.8) | 0.98 (0.95 to 1.02) |
| 50-54 | 11096 | 261 (256 - 265) |  | 10802 | 249 (245 - 254) |  | -294 | -11.1 (-17.9 to -4.4) | 0.96 (0.93 to 0.98) |
| 55-59 | 16195 | 380 (374 - 386) |  | 15588 | 371 (365 - 376) |  | -607 | -9.7 (-18.0 to -1.4) | 0.97 (0.95 to 1.00) |
| 60-64 | 19261 | 535 (527 - 542) |  | 19894 | 523 (516 - 530) |  | 633 | -11.9 (-22.4 to -1.5) | 0.98 (0.96 to 1.00) |
| 65-69 | 20181 | 826 (814 - 837) |  | 21201 | 804 (794 - 815) |  | 1020 | -21.4 (-37.1 to -5.7) | 0.97 (0.96 to 0.99) |
| 70-74 | 26083 | 1,371 (1,355 - 1,388) |  | 26466 | 1,323 (1,307 - 1,339) |  | 383 | -48.3 (-71.4 to -25.3) | 0.96 (0.95 to 0.98) |
| 75-79 | 42208 | 2,633 (2,608 - 2,658) |  | 41835 | 2,610 (2,585 - 2,635) |  | -373 | -22.3 (-57.8 to 13.1) | 0.99 (0.98 to 1.01) |
| 80-84 | 53140 | 5,031 (4,989 - 5,074) |  | 55154 | 4,965 (4,923 - 5,006) |  | 2014 | -66.7 (-126.2 to -7.1) | 0.99 (0.98 to 1.00) |
| 85+ | 85533 | 11,994 (11,914 - 12,074) |  | 93202 | 11,876 (11,800 - 11,952) |  | 7669 | -118.1 (-228.9 to -7.3) | 0.99 (0.98 to 1.00) |
| Men |  |  |  |  |  |  |  |  |  |
| 0-4 | 587 | 60 (55 - 65) |  | 493 | 55 (50 - 59) |  | -94 | -5.4 (-12.2 to 1.5) | 0.91 (0.81 to 1.03) |
| 5-9 | 108 | 9 (7 - 11) |  | 80 | 7 (5 - 8) |  | -28 | -2.3 (-4.5 to 0.0) | 0.75 (0.56 to 1.00) |
| 10-14 | 108 | 9 (7 - 11) |  | 121 | 10 (8 - 12) |  | 13 | 1.1 (-1.4 to 3.6) | 1.12 (0.86 to 1.45) |
| 15-19 | 374 | 26 (24 - 29) |  | 365 | 28 (25 - 30) |  | -9 | 1.2 (-2.7 to 5.1) | 1.04 (0.90 to 1.21) |
| 20-24 | 682 | 39 (36 - 42) |  | 650 | 38 (35 - 41) |  | -32 | -0.8 (-4.9 to 3.4) | 0.98 (0.88 to 1.09) |
| 25-29 | 938 | 52 (49 - 55) |  | 1010 | 55 (51 - 58) |  | 72 | 2.7 (-2.0 to 7.4) | 1.05 (0.96 to 1.15) |
| 30-34 | 1132 | 70 (66 - 74) |  | 1108 | 68 (64 - 72) |  | -24 | -1.3 (-7.0 to 4.4) | 0.98 (0.90 to 1.07) |
| 35-39 | 1990 | 99 (94 - 103) |  | 1860 | 96 (92 - 101) |  | -130 | -2.4 (-8.6 to 3.8) | 0.98 (0.92 to 1.04) |
| 40-44 | 2755 | 141 (135 - 146) |  | 2702 | 138 (133 - 143) |  | -53 | -2.9 (-10.3 to 4.5) | 0.98 (0.93 to 1.03) |
| 45-49 | 5328 | 234 (228 - 241) |  | 5035 | 226 (220 - 233) |  | -293 | -8.0 (-16.8 to 0.9) | 0.97 (0.93 to 1.00) |
| 50-54 | 8096 | 377 (369 - 385) |  | 7728 | 354 (346 - 362) |  | -368 | -23.3 (-34.7 to -11.9) | 0.94 (0.91 to 0.97) |
| 55-59 | 11942 | 559 (549 - 569) |  | 11443 | 541 (531 - 551) |  | -499 | -17.9 (-32.0 to -3.8) | 0.97 (0.94 to 0.99) |
| 60-64 | 14219 | 800 (787 - 813) |  | 14534 | 774 (761 - 786) |  | 315 | -26.2 (-44.4 to -8.0) | 0.97 (0.95 to 0.99) |
| 65-69 | 14417 | 1,224 (1,204 - 1,244) |  | 15240 | 1,198 (1,179 - 1,217) |  | 823 | -25.6 (-53.2 to 2.0) | 0.98 (0.96 to 1.00) |
| 70-74 | 17633 | 1,995 (1,966 - 2,025) |  | 18128 | 1,940 (1,912 - 1,969) |  | 495 | -54.9 (-95.7 to -14.1) | 0.97 (0.95 to 0.99) |
| 75-79 | 25443 | 3,765 (3,718 - 3,811) |  | 25521 | 3,742 (3,696 - 3,787) |  | 78 | -23.2 (-88.3 to 42.0) | 0.99 (0.98 to 1.01) |
| 80-84 | 27070 | 6,999 (6,916 - 7,082) |  | 28418 | 6,875 (6,795 - 6,955) |  | 1348 | -124.4 (-239.9 to -8.9) | 0.98 (0.97 to 1.00) |
| 85+ | 27500 | 14,439 (14,269 - 14,610) |  | 30727 | 14,332 (14,171 - 14,492) |  | 3227 | -107.8 (-341.9 to 126.3) | 0.99 (0.98 to 1.01) |
| Women |  |  |  |  |  |  |  |  |  |
| 0-4 | 468 | 50 (46 - 55) |  | 366 | 43 (38 - 47) |  | -102 | -7.6 (-14.0 to -1.3) | 0.85 (0.74 to 0.97) |
| 5-9 | 70 | 6 (5 - 8) |  | 62 | 6 (4 - 7) |  | -8 | -0.7 (-2.7 to 1.3) | 0.89 (0.63 to 1.25) |
| 10-14 | 76 | 7 (5 - 8) |  | 82 | 7 (6 - 9) |  | 6 | 0.5 (-1.7 to 2.7) | 1.07 (0.79 to 1.47) |
| 15-19 | 236 | 18 (16 - 20) |  | 198 | 16 (14 - 18) |  | -38 | -1.9 (-5.1 to 1.3) | 0.89 (0.74 to 1.08) |
| 20-24 | 444 | 28 (25 - 30) |  | 468 | 30 (27 - 33) |  | 24 | 2.1 (-1.6 to 5.9) | 1.08 (0.94 to 1.22) |
| 25-29 | 497 | 31 (28 - 33) |  | 578 | 35 (32 - 38) |  | 81 | 4.3 (0.4 to 8.2) | 1.14 (1.01 to 1.29) |
| 30-34 | 656 | 43 (40 - 46) |  | 644 | 43 (39 - 46) |  | -12 | -0.3 (-5.0 to 4.4) | 0.99 (0.89 to 1.11) |
| 35-39 | 1127 | 58 (55 - 61) |  | 1147 | 62 (58 - 66) |  | 20 | 3.9 (-1.1 to 8.8) | 1.07 (0.98 to 1.16) |
| 40-44 | 1464 | 77 (73 - 81) |  | 1453 | 76 (73 - 80) |  | -11 | -0.7 (-6.3 to 4.8) | 0.99 (0.92 to 1.06) |
| 45-49 | 2373 | 108 (103 - 112) |  | 2384 | 111 (106 - 115) |  | 11 | 2.9 (-3.3 to 9.1) | 1.03 (0.97 to 1.09) |
| 50-54 | 3000 | 142 (137 - 147) |  | 3074 | 143 (138 - 148) |  | 74 | 1.1 (-6.1 to 8.3) | 1.01 (0.96 to 1.06) |
| 55-59 | 4253 | 200 (194 - 206) |  | 4145 | 198 (192 - 204) |  | -108 | -2.2 (-10.7 to 6.3) | 0.99 (0.95 to 1.03) |
| 60-64 | 5042 | 277 (269 - 284) |  | 5360 | 278 (271 - 286) |  | 318 | 1.8 (-8.9 to 12.5) | 1.01 (0.97 to 1.05) |
| 65-69 | 5764 | 455 (444 - 467) |  | 5961 | 437 (426 - 448) |  | 197 | -18.2 (-34.4 to -2.1) | 0.96 (0.93 to 1.00) |
| 70-74 | 8450 | 830 (812 - 847) |  | 8338 | 782 (765 - 799) |  | -112 | -47.8 (-72.2 to -23.4) | 0.94 (0.91 to 0.97) |
| 75-79 | 16765 | 1,808 (1,780 - 1,835) |  | 16314 | 1,772 (1,745 - 1,799) |  | -451 | -35.5 (-74.1 to 3.0) | 0.98 (0.96 to 1.00) |
| 80-84 | 26070 | 3,895 (3,847 - 3,942) |  | 26736 | 3,833 (3,787 - 3,879) |  | 666 | -61.6 (-127.5 to 4.3) | 0.98 (0.97 to 1.00) |
| 85+ | 58033 | 11,103 (11,013 - 11,193) |  | 62475 | 10,953 (10,867 - 11,039) |  | 4442 | -150.1 (-274.7 to -25.4) | 0.99 (0.98 to 1.00) |
