# Supplementary material for: Cause-specific mortality in Korea during the first year of the COVID-19 pandemic
Source: Epidemiol Health. 2022 Nov 23;44:e2022110. doi: 10.4178/epih.e2022110 (PMC10106553; doi:10.4178/epih.e2022110)
Supplement: Supplementary file 7 [file epih-44-e2022110-Supplementary-7.docx]

Supplementary Material 7. The age-specific mortality rate (per 100,000) among Korean women aged 25-29 according to sub-specific causes of death between 2019 and 2020

|  | 2019 | 2020 | Rate Difference |
| --- | --- | --- | --- |
| **Certain infectious and parasitic diseases(A00-B99, U07.1, U07.2, U08-U10)** |  |  |  |
| Tuberculosis (A15-A19) | 0.06 | 0.06 | 0.00 |
| Sepsis (A40-A41) | 0.12 | 0.18 | 0.06 |
| Covid19 (U07.1, U07.2, U08-U10) | 0.00 | 0.00 | 0.00 |
| Others | 0.06 | 0.24 | 0.18 |
| **Malignant neoplasms(C00-C97)** |  |  |  |
| Oesophageal cancer (C15) | 0.00 | 0.00 | 0.00 |
| Stomach cancer (C16) | 0.62 | 0.79 | 0.17 |
| Colorectal cancer (C18-C21) | 0.19 | 0.36 | 0.18 |
| Liver cancer (C22) | 0.31 | 0.24 | -0.07 |
| Gallbladder cancer (C23) | 0.00 | 0.00 | 0.00 |
| Biliary tract cancer (C24) | 0.00 | 0.12 | 0.12 |
| Pancreatic cancer (C25) | 0.06 | 0.06 | 0.00 |
| Lung cancer (C33-C34) | 0.19 | 0.12 | -0.06 |
| Breast cancer (C50), Female | 0.49 | 0.61 | 0.11 |
| Cervical cancer (C53) | 0.31 | 0.18 | -0.13 |
| Uterus cancer (C54-C55) | 0.00 | 0.06 | 0.06 |
| Ovarian cancer (C56) | 0.19 | 0.24 | 0.06 |
| Prostate cancer (C61) | 0.00 | 0.00 | 0.00 |
| Kidney cancer (C64) | 0.00 | 0.06 | 0.06 |
| Bladder cancer (C67) | 0.00 | 0.00 | 0.00 |
| Brain cancer (C70-72) | 0.31 | 0.54 | 0.24 |
| Non-Hodgkin's lymphoma (C82-C86) | 0.31 | 0.06 | -0.25 |
| Multiple myeloma (C90) | 0.00 | 0.00 | 0.00 |
| Leukaemia (C91-C95) | 0.80 | 0.97 | 0.17 |
| Other neoplasms(D00-D48) | 1.11 | 0.91 | -0.20 |
| **Endocrine, nutritional and metabolic diseases(E00-E90)** |  |  |  |
| Diabetes mellitus (E10-E14) | 0.25 | 0.24 | 0.00 |
| Others | 0.19 | 0.18 | 0.00 |
| **Mental and behavioural disorders and nervous system diseases(F00-F99, G00-G99)** |  |  |  |
| Dementia (F00-F03, G30) | 0.00 | 0.00 | 0.00 |
| Alcoholism (F10) | 0.12 | 0.06 | -0.06 |
| Parkinson's disease (G20) | 0.00 | 0.00 | 0.00 |
| Others | 0.93 | 0.67 | -0.26 |
| **Diseases of the circulatory system(I00-I99)** |  |  |  |
| Hypertensive diseases (I10-I15) | 0.00 | 0.00 | 0.00 |
| Ischaemic heart diseases (I20-I25) | 0.19 | 0.30 | 0.12 |
| Atrial fibrillation (I48) | 0.00 | 0.00 | 0.00 |
| Heart failure (I50) | 0.06 | 0.06 | 0.00 |
| Cerebrovascular diseases (I60-I69) | 0.62 | 0.61 | -0.01 |
| Haemorrhagic stroke (I60-I62) | 0.49 | 0.54 | 0.05 |
| Ischaemic stroke (I63) | 0.06 | 0.06 | 0.00 |
| Other stroke (I64-I69) | 0.06 | 0.00 | -0.06 |
| Aortic aneurysm (I71) | 0.06 | 0.00 | -0.06 |
| Others | 0.49 | 0.79 | 0.29 |
| **Diseases of the respiratory system(J00-J99)** |  |  |  |
| Pneumonia (J12-J18) | 0.31 | 0.18 | -0.13 |
| Chronic lower respiratory diseases (J40-J47) | 0.00 | 0.06 | 0.06 |
| Pneumonitis due to solids and liquids (J69) | 0.06 | 0.06 | 0.00 |
| Interstitial pulmonary diseases (J84) | 0.00 | 0.00 | 0.00 |
| Others | 0.06 | 0.00 | -0.06 |
| **Diseases of the digestive system(K00-K93)** |  |  |  |
| Liver diseases (K70-K77) | 0.62 | 0.67 | 0.05 |
| Alcoholic liver disease (K70) | 0.31 | 0.61 | 0.30 |
| Liver cirrhosis (K74) | 0.06 | 0.00 | -0.06 |
| Others | 0.19 | 0.30 | 0.12 |
| **Diseases of the skin and subcutaneous tissue(L00-L99)** |  |  |  |
| **Diseases of the musculoskeletal system and connective tissue(M00-M99)** |  |  |  |
| **Diseases of the genitourinary system(N00-N99)** |  |  |  |
| Renal failure (N17-N19) | 0.00 | 0.06 | 0.06 |
| Others | 0.00 | 0.00 | 0.00 |
| **Pregnancy, childbirth, and the puerperium (O00-O99)** |  |  |  |
| **Certain conditions originating in the perinatal period(P00-P96)** |  |  |  |
| **Congenital malformations, deformations, and chromosomal abnormalities(Q00-Q99)** |  |  |  |
| **Symptoms, signs, and abnormal clinical and laboratory findings, NEC(R00-R99)** |  |  |  |
| Senility (R54) | 0.00 | 0.00 | 0.00 |
| Other ill-defined and unspecified causes of mortality (R99) | 0.86 | 1.09 | 0.22 |
| Others | 0.31 | 0.36 | 0.05 |
| **External causes of morbidity and mortality(V01-Y98)** |  |  |  |
| Transport Accidents (V01-V99) | 0.93 | 1.51 | 0.59 |
| Falls (W00-W19) | 0.25 | 0.30 | 0.06 |
| Intentional self-harm (X60-X84) | 16.55 | 19.43 | 2.88 |
| Others | 1.54 | 1.27 | -0.27 |
| **Other causes** | 6.30 | 6.78 | 0.48 |
